# GLSP mitigates vascular aging by promoting Sirt7-mediated Keap1 deacetylation and Keap1-Nrf2 dissociation

**DOI:** 10.7150/thno.110324

**Published:** 2025-03-18

**Authors:** Yanfei Cheng, Guobin Zheng, Heming Huang, Jingyu Ni, Yun Zhao, Yuting Sun, Yingxin Chang, Shangjing Liu, Feng He, Dan Li, Yuying Guo, Yaodong Miao, Mengxin Xu, Dongyue Wang, Yunsha Zhang, Yunqing Hua, Shu Yang, Guanwei Fan, Chuanrui Ma

**Affiliations:** 1 First Teaching Hospital of Tianjin University of Traditional Chinese Medicine, National Clinical Research Center for Chinese Medicine Acupuncture and Moxibustion, Tianjin, China.; 2 NHC Key Lab of Hormones and Development and Tianjin Key Lab of Metabolic Diseases, Tianjin Medical University Chu Hsien-I Memorial Hospital & Institute of Endocrinology, Tianjin 300134, China.; 3 Department of Geriatrics, Peking University Shenzhen Hospital, Shenzhen, China.; 4 Department of Geriatrics, Shenzhen People's Hospital (The Second Clinical Medical College, Jinan University; The First Affiliated Hospital, Southern University of Science and Technology), Shenzhen, Guangdong 518020, China.; 5 Second Affiliated Hospital of Tianjin University of Traditional Chinese Medicine, Tianjin, China.; 6 Culture and Industry Research Center of Li Shizhen Traditional Chinese Medicine, Li Shizhen College of Traditional Chinese Medicine, Huanggang Normal University, Huanggang, 438000, China.

**Keywords:** vascular aging, vascular calcification, atherosclerosis, GLSP, Sirt7, Nrf2, deacetylation.

## Abstract

**Background and Purpose:** Vascular aging is a prior marker of human aging and a significant contributor to atherosclerosis and vascular calcification. However, there are limited pharmacological options available to mitigate vascular aging. Thus, understanding the mechanisms underlying vascular aging and age-related atherosclerosis and vascular calcification is crucial. This study investigates the targets of vascular aging and elucidates the role and mechanisms of *Ganoderma lucidum* spore powder (GLSP) in mitigating vascular aging and aging-associated atherosclerosis as well as vascular calcification.

**Methods:** The anti-vascular aging effects of GLSP was determined in aged C57BL/6J mice and the targets of GLSP was identified through transcriptome sequencing. Additionally, the protective effects of GLSP on the aged vasculature were assessed by examining atherosclerosis in apoE^-/-^ mice and vascular calcification in VD_3_ and nicotine-induced mice. *In vitro*, the protective effects of GLSP triterpenes against vascular aging and calcification was determined in vascular smooth muscle cells (VSMCs).

**Results:** GLSP exerted anti-vascular aging effects by regulating the cell cycle and senescence-associated secretory phenotype (SASP), mitigating DNA damage, reducing oxidative stress, improving mitochondrial function and modulating metabolic levels. Furthermore, GLSP improved vascular aging-associated atherosclerosis and vascular calcification *in vivo*. Mechanistically, RNA sequencing revealed an upregulation of Sirt7 expression after GLSP treatment. Sirt7 inhibitor exacerbated VSMCs senescence and calcification in senescent VSMCs and abolished the anti-senescence and the inhibitory effect of GLSP triterpenes on VSMCs senescence and calcification. Innovatively, we found that Sirt7 interacted with Keap1 and facilitated Keap1 deacetylation, which promoted Keap1-Nrf2 dissociation and consequently enhanced Nrf2 nuclear translocation and activation.

**Conclusion:** GLSP alleviates vascular aging by exerting antioxidant effects through the activation of the Sirt7-Nrf2 axis, providing a promising new strategy for delaying vascular aging, atherosclerosis and vascular calcification.

## Introduction

In epidemiological studies, age is the most important cardiovascular risk factor 1. Age-related pathological alterations of the vasculature, such as cardiovascular diseases, result in high morbidity and mortality rates worldwide 2, 3. Vascular aging is a multifaceted pathological process, distinguished by both structural and functional alterations in the vasculature, such as thickening of elastic arterial walls, dilation of the lumen, heightened endothelial permeability, calcification of the tunica media, along with an accumulation of collagen and the fragmentation of elastic fibers, which serves as a critical trigger for the development and progression of cardiovascular disease by contributing atherosclerosis and vascular calcification 4-8. No effective therapeutic strategies are currently available to prevent or halt the progression of vascular aging.

As we age, the decreased efficiency of the respiratory chain contributes to the increased electron leakage and subsequently higher reactive oxygen species (ROS) production, which lead oxidative damage to mitochondrial DNA and cellular DNA, and consequently result in mitochondrial dysfunction and cell senescence 9-11. Senescent vascular cells lead to vascular aging and exacerbate the occurrence of adverse cardiovascular events 1, 10, 12, 13. Aged vascular are marked by heightened vascular stiffness and endothelial dysfunction, alongside diminished proliferative and migratory capacities of both vascular endothelial cells and VSMCs 14, 15. Senescent vascular endothelial cells and VSMCs secrete senescence-associated pro-inflammatory cytokines, including inflammatory factors, chemokines, matrix metalloproteinases and vascular adhesion molecules, which promote the transition of normal cells into senescent cells and promote the progression of vascular aging 16-19.

Atherosclerosis and vascular calcification are hallmarks of vascular aging. Vascular calcification is a pathological process characterized by abnormal deposition of calcium and phosphate crystals in the vascular wall, which promote the risk of plaque rupture 20, 21. Calcification has been determined to be an active process driven in part by VSMC osteogenic differentiation within the vascular wall 22. In senescent VSMCs, elevated ROS not only leads to a loss of contractile capacity but also upregulates the expression of the osteogenic transcription factor RUNX2, concurrently, downregulates markers of VSMC, thereby facilitating their differentiation toward an osteogenic phenotype 23. Inflammatory factors secreted by senescent cells can also activate the expression of the osteogenic marker BMP2 in vascular cells, promoting the transition of VSMC to osteoblast-like cells and contributing to vascular calcification 24, 25. In addition, VSMC senescence not only results in a decreased VSMC content within plaques but also elevates levels of collagen-degrading matrix metalloproteinases, collectively exacerbating plaque instability and accelerating the progression of atherosclerosis 26, 27. Moreover, senescent macrophages are deleterious at all stages of atherosclerosis. Senescent macrophages accumulate in the subendothelial space at the onset of atherosclerosis, where they drive pathology by increasing expression of atherogenic and inflammatory cytokines and chemokines 28. Therefore, mitigating vascular aging is a therapeutic target in cardiovascular diseases and reducing oxidative stress and inflammation is an important strategy for slowing down vascular aging.

*Ganoderma lucidum* is a traditional Chinese medicine with a variety of pharmacological effects, including anti-inflammatory 29-31, antioxidant 32, 33, energy metabolism regulation and immunomodulation 34. GLSP is extracted from the germ cells of *Ganoderma lucidum.* Previously, we found that GLSP mitigated the atherosclerosis and vascular calcification in LDLR^-/-^ mice 35. The above pharmacological studies indicated that GLSP possessed the anti-aging potential. Nonetheless, the precise effects and underlying mechanisms by which GLSP mitigated vascular aging remain unknown. This study delved into the protective role of GLSP in combating vascular aging. The findings of the study suggested that GLSP mitigated vascular aging by not only improving cell cycle arrest and diminishing DNA damage, but also by suppressing inflammation and oxidative stress. Mechanistically, GLSP mitigated vascular aging, atherosclerosis and vascular calcification by upregulating Sirt7 expression. Sirt7 enhanced the deacetylation of Keap1 and promoted its dissociation from Nrf2. This process induced Nrf2 nuclear translocation and activated downstream antioxidant genes. In summary, GLSP exerted its protective effects by diminishing oxidative stress and inflammation, ultimately mitigating vascular aging as well as the related atherosclerosis and vascular calcification. GLSP may act as a novel therapeutic strategy for addressing vascular aging, and age-associated atherosclerosis and vascular calcification.

## Results

### GLSP improved vascular aging and remodeling in aged mice

**Figure 1:**
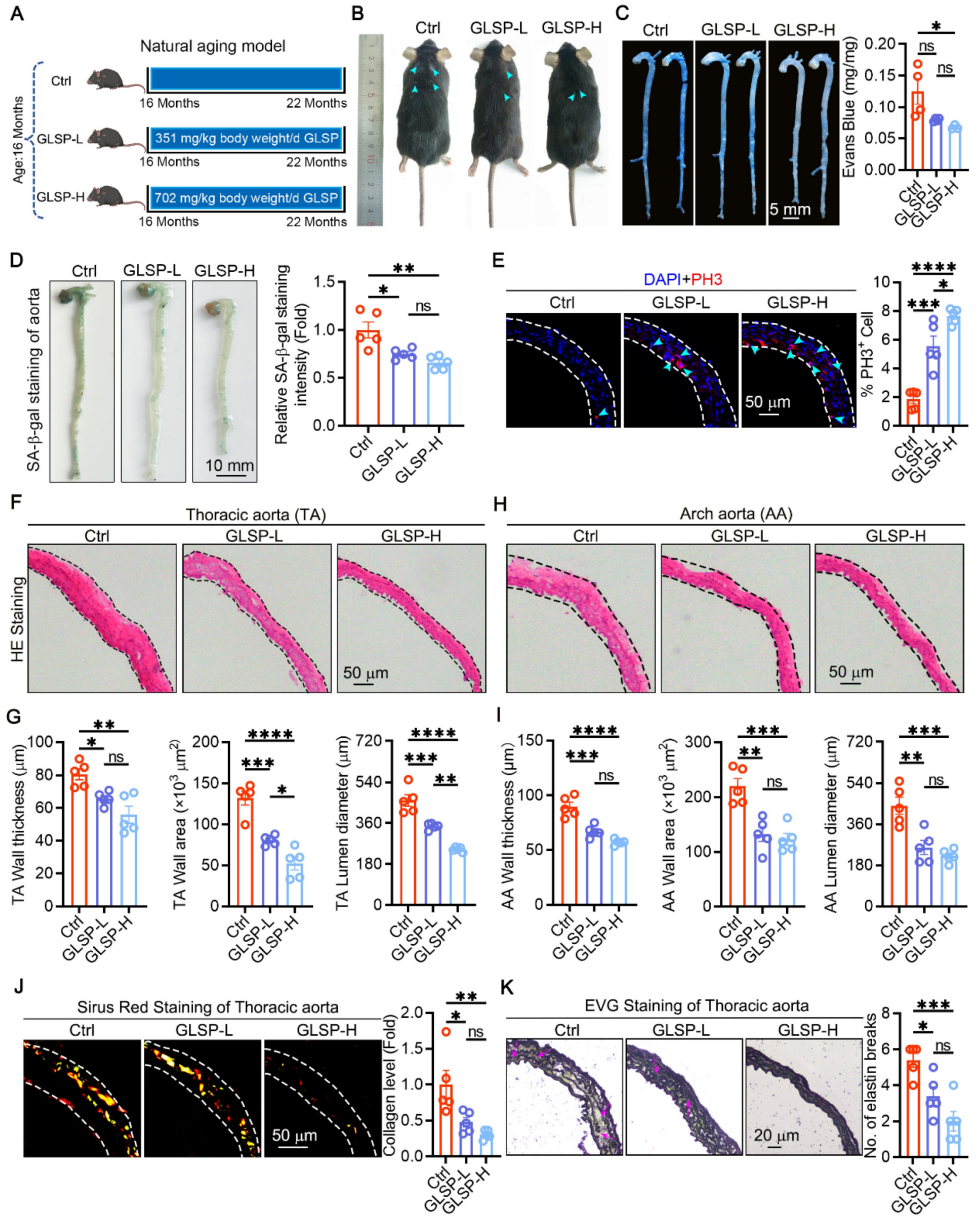
** The alleviating effects of GLSP on vascular aging. (A)** Grouping of naturally aged mice (n = 5). **(B)** A photo of the back fur of naturally aging mice. **(C)** Evans blue staining was used to detect vascular permeability (n = 4).** (D)** Assess the degree of aortic aging through senescence-associated β-galactosidase staining (n = 5).** (E)** PH3 immunofluorescence staining was used to assess cell proliferation (n = 5).** (F-I)** The thoracic aorta and aortic arch were stained with HE staining, followed by quantitative analysis of vascular wall thickness, wall area and lumen diameter (n = 5). **(J)** Sirius Red staining on the thoracic aorta was performed to detect collagen content (n = 5). **(K)** The degree of elastic fiber fracture was detected by EVG staining (n = 5). *P < 0.05, **P < 0.01, ***P < 0.001, ****P < 0.0001. All experiments were compared with the Ctrl group and error bars denote SEM. Ctrl: Control group.

To investigate the effect of GLSP on vascular aging, we used a natural aging mice model and then treated with GLSP for 6 months **(Figure 1A)**. There were no significant changes in the body weight and organ indices of the mice **(Figure S1A-C)**, nor were there notable alterations in the serum levels of TG, TC and HDL, with the exception of LDL **(Figure S1D)**. Furthermore, from the photos of the mice's backs, the mice in the Ctrl group developed whitening and shedding of their fur, which was effectively alleviated by GLSP **(Figure 1B)**. Increased vascular permeability is a marker of vascular aging 36. Therefore, we further used Evans blue staining to detect vascular permeability in aged mice, and found that GLSP significantly reduced vascular permeability compared to the Ctrl group **(Figure 1C)**. Senescent cell accumulation is a significant feature of aging 37. Senescence-associated beta galactosidase staining was used to detect the degree of aortic aging, and it was found that GLSP intervention significantly reduced the number of senescent cells **(Figure 1D)**. Senescent cells typically exhibit a decreased proliferative capacity 38, 39. By detecting the G2-M phase marker protein PH3, we found that GLSP significantly increased the number of PH3-positive cells, indicating that GLSP can promote cell proliferation (**Figure 1E**). Moreover, the process of aging is associated with the onset of pathological vascular remodeling 40, 41. Histological examination via HE staining revealed that administration of GLSP was effective in reducing vascular wall thickness, vascular wall area, and vascular lumen diameter **(Figure 1F-I)**. Vascular aging is accompanied by extracellular matrix remodeling, causing changes in the composition of elastin and collagen in large elastic arteries 42. We evaluated the collagen content in the vascular using Sirius Red staining, and found that GLSP was capable to reduce the deposition of collagen in aorta **(Figure 1J)**. Elastic fiber rupture serves as a key hallmark of vascular aging 10. Therefore, we used EVG staining to detect the degree of elastic fiber breakage, and observed that GLSP was able to decrease the breakage of elastic fibers **(Figure 1K)**. These findings suggested that GLSP can alleviate vascular aging by improving pathological vascular remodeling.

### GLSP attenuated vascular aging by regulating cell cycle and senescence-associated secretory phenotype

**Figure 2:**
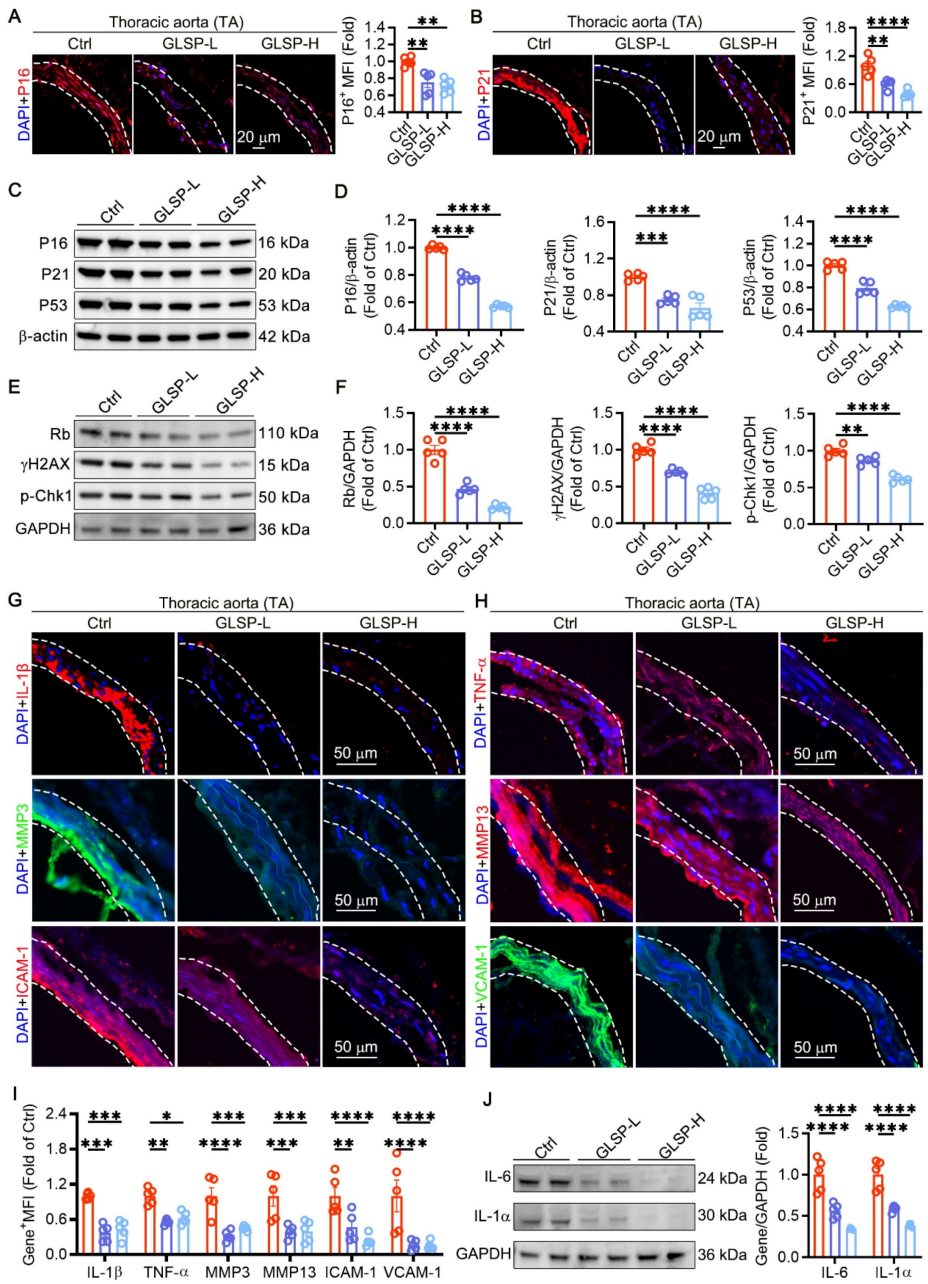
** Cell cycle and senescence-associated secretory phenotype can be regulated by GLSP. (A, B)** Immunofluorescence staining and quantitative analysis of cell cycle regulatory genes P16 and P21 (n = 5). **(C-F)** Immunoblotting analysis of the expression levels of P16, P21, P53, Rb, γH2AX and p-Chk1 in the aortas of mice from each group (n = 5). **(G-I)** Immunofluorescence staining and quantitative analysis of SASP factors IL-1β, TNF-α, MMP3, MMP13, ICAM-1 and VCAM-1 (n = 5). **(J)** Western blot analysis was performed to detect the protein expression of IL-6 and IL-1α in the aorta (n = 5). *P < 0.05, **P < 0.01, ***P < 0.001, ****P < 0.0001. All experiments were compared with the Ctrl group and error bars denote SEM.

During the process of vascular aging, an increase in the production of ROS promotes the DNA damage response of vascular endothelial cells and VSMCs, impairs DNA replication ability, and ultimately permanently exits the cell cycle 1, 10, 43. This indicates that regulating the cell cycle is crucial for delaying vascular aging. Therefore, we detected key genes P16 and P21 that regulated the cell cycle pathway in vasculature. The results showed that GLSP significantly reduced the expression levels of P16 and P21 **(Figure 2A-B)**. Furthermore, the western blot analysis revealed that GLSP significantly attenuated the protein expression levels of P16, P21 and P53 **(Figure 2C-D)**. Phosphorylation of H2AX and Chk1 is widely recognized as an indicator of DNA double-strand breaks, whereas Rb plays a pivotal role in cell cycle regulation 44, 45. Through immunoblotting analysis of aortic tissues, we observed that GLSP markedly diminished the phosphorylation of H2AX and Chk1 while simultaneously attenuating the expression of Rb **(Figure 2E-F)**. Senescent cells have a complex SASP, which can secrete a series of inflammatory factors, matrix metalloproteinases, and intercellular adhesion factors through autocrine or paracrine secretion, thereby promoting the aging of other cells 46. In our study, we observed that GLSP led to a significant reduction in the expression levels of IL-1β and TNF-α, thereby demonstrating pronounced anti-inflammatory effects. Additionally, GLSP notably diminished the expression of matrix metalloproteinases MMP3 and MMP13. Furthermore, GLSP also attenuated the expression of intercellular adhesion molecules ICAM-1 and VCAM-1 **(Figure 2G-I)**. Intriguingly, subsequent to GLSP intervention, there was a notable reduction in IL-6 and IL-1α levels **(Figure 2J)**. Moreover, ELISA analysis of serum inflammatory factors revealed that GLSP significantly decreased the secretion of TNF-α and IL-1β **(Figure S2A-B)**. In conclusion, GLSP effectively alleviated cell cycle blockade and SASP and thereby ameliorating vascular aging.

### GLSP reduced oxidative stress in aged aorta by ameliorating mitochondrial dysfunction and enhancing mitophagy

**Figure 3:**
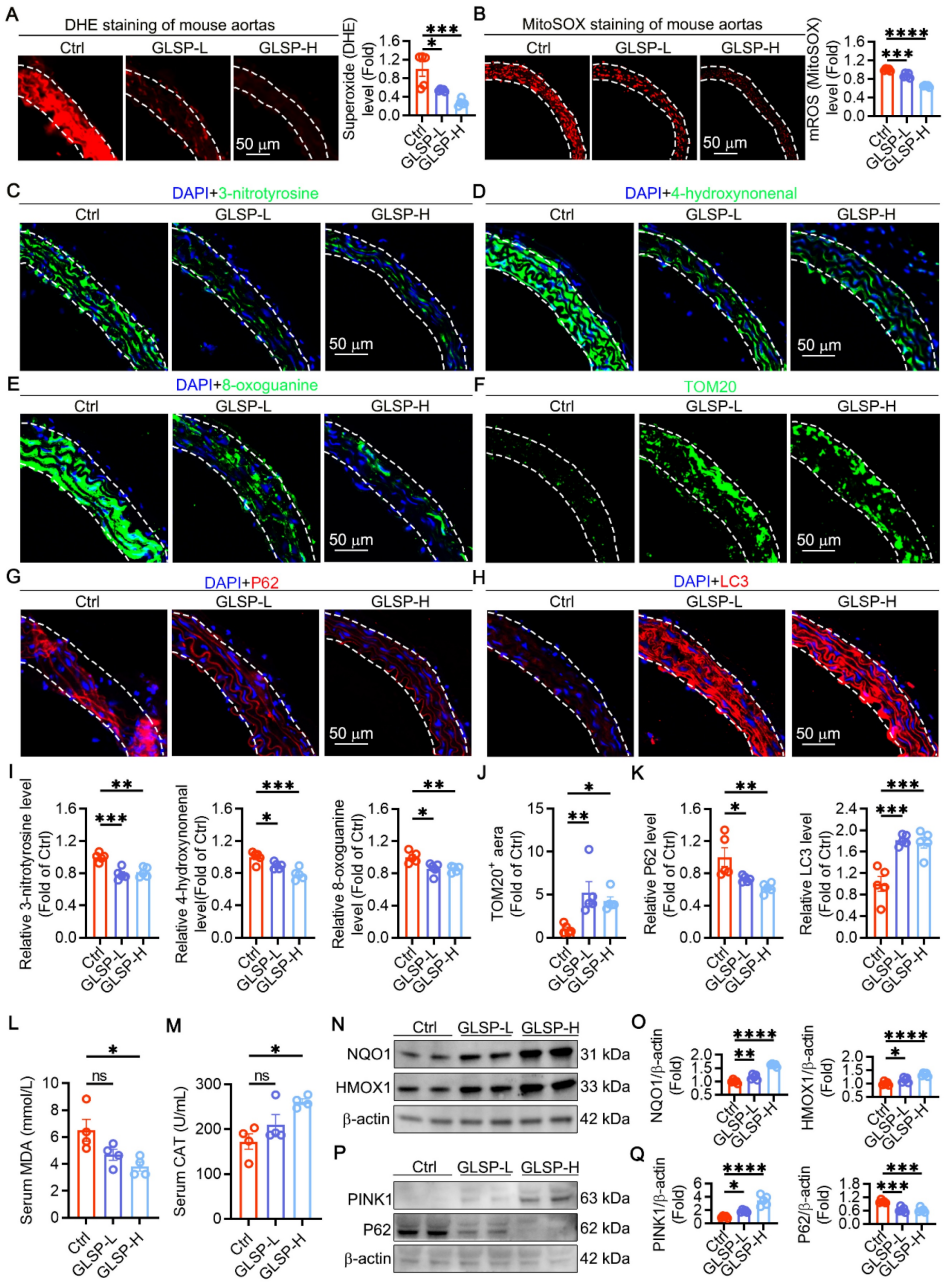
** GLSP improved mitochondrial function and mitophagy and reduced oxidative stress in vascular aging. (A)** Detection of aortic total superoxide levels by dihydroethidium fluorescent probe (n = 5). **(B)** MitoSOX staining for detection of mitochondrial ROS (n = 5). **(C-E)** Immunofluorescence detection of protein oxidation, lipid peroxidation and oxidative DNA damage levels in thoracic aorta (n = 5). **(F)** Detection of mitochondrial content in aorta by TOM20 staining (n = 5). **(G, H)** Assessment of autophagy flux by immunofluorescence detection of P62 and LC3 protein expression level (n = 5)**. (I)** The fluorescence quantification of 3-nitrotyrosine, 4-hydroxynonenal, 8-oxoguanine (n = 5). **(J)** Statistical analysis of TOM20-positive fluorescent area (n = 5). **(K)** The fluorescence quantification of P62, LC3 (n = 5). **(L, M)** Serum MDA and CAT levels (n = 4). **(N, O)** Immunoblotting revealed the expression levels of NQO1 and HMOX1 (n = 5). **(P, Q)** Immunoblotting revealed the expression levels of PINK1 and P62 (n = 5). *P < 0.05, **P < 0.01, ***P < 0.001, ****P < 0.0001. All experiments were compared with the Ctrl group and error bars denote SEM.

Oxidative stress is an important factor in the vascular aging process 9. Increased ROS levels cause damage to mitochondria and biomacromolecules, impairing cellular function and promoting the onset and progression of vascular aging 47. We examined the total superoxide level in the aorta by DHE staining and found that GLSP could significantly reduce the total ROS levels in the aorta **(Figure 3A)**. Then we evaluated mitochondrial ROS with MitoSOX probe, and GLSP could significantly reduce mitochondrial ROS levels (**Figure 3B**). The oxidative stress in tissues and cells promotes oxidative damage to macromolecules, such as protein, lipid and DNA damage in the aorta 40. Therefore, we examined the regulatory effects of GLSP on protein oxidation, lipid peroxidation, and DNA oxidative damage and found that GLSP significantly reduced the expression levels of the above markers **(Figure 3C-E,I)**. During aging, the number of mitochondria undergoes significant changes 48, 49. Using TOM20 staining to localize mitochondria, we found that GLSP could significantly increase mitochondrial quantity **(Figure 3F, J)**. Mitochondria in cells regulate organismal health and longevity through multiple pathways. Mitochondrial autophagy has also been shown to be critical for maintaining organismal health and preventing aging-related degeneration 50, 51. We examined the expression of autophagy-related proteins P62 and LC3 protein levels in aortas by immunofluorescence, the results showed that GLSP could significantly increase mitophagy levels **(Figure 3G-H, K)**. In addition, GLSP intervention significantly reduced the levels of MDA in the serum and increased the levels of CAT **(Figure 3L-M)**. NQO1 and HMOX1, functioning as pivotal antioxidant genes, are instrumental in preserving redox equilibrium and substantially reducing oxidative damage within the organism 52, 53. Western blot analysis revealed that GLSP treatment led to an increased expression of both proteins **(Figure 3N-O)**. Additionally, immunoblotting experiments revealed that GLSP significantly increased the expression of PINK1 and decreased the expression of the P62 protein **(Figure 3P-Q)**. In summary, GLSP mitigated vascular aging by reducing oxidative stress levels in aged aorta, enhancing mitophagy and thereby improving mitochondrial function.

### GLSP attenuated atherosclerosis and aged markers in the advanced atherosclerotic apoE^-/-^ mice

**Figure 4:**
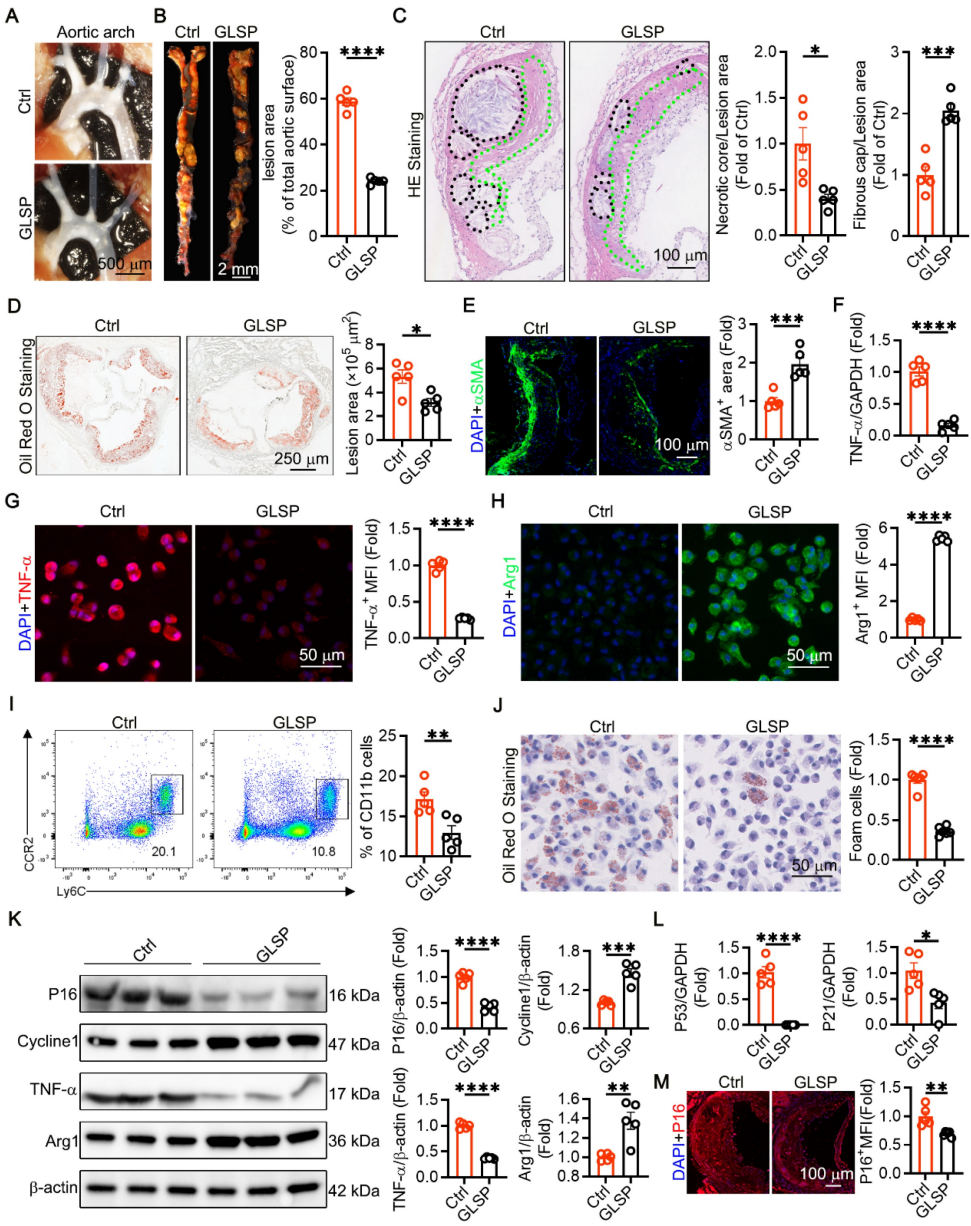
** GLSP attenuated advanced atherosclerosis and aged markers in aorta. (A)** Representative *in situ* images of atherosclerotic plaque in the aortic arch. **(B)** Oil red O staining showed the whole aortic plaque area (n = 5). **(C)** HE staining showed necrotic core and fibrous cap area (n = 5). **(D)** Oil red O staining showed aortic root plaque area (n = 5). **(E)** Representative immunofluorescence images of αSMA in the aortic root (n = 5). **(F)** qRT-PCR analysis of the expression of TNF-α in primary peritoneal macrophages of apoE^-/-^ mice (n = 5). **(G, H)** Immunofluorescence staining showed the expression of TNF-α, and Arg1 in primary peritoneal macrophages (n = 5). **(I)** Flow cytometry analysis of the proportion of pro-inflammatory monocytes in peripheral blood (n = 5). **(J)** Representative Oil Red O staining images of primary peritoneal macrophages (n = 5). **(K)** Quantification of P16, Cycline1, TNF-α and Arg1 protein expression via western blotting in peritoneal macrophages from apoE^-/-^ mice (n = 5).** (L)** qRT-PCR analysis of the expression of P53 and P21 in primary peritoneal macrophages of apoE^-/-^ mice (n = 5). **(M)** Representative immunofluorescence images of P16 in the aortic root (n = 5). *P < 0.05, **P < 0.01, ***P < 0.001, ****P < 0.0001. All experiments were compared with the Ctrl group and error bars denote SEM.

Atherosclerosis is classed as a disease of aging 4. Senescent endothelial and VSMCs promote the development of advanced atherosclerosis 4, 54, 55. We constructed a model of advanced atherosclerosis using apoE**^-/-^** mice. The results showed that the GLSP group mice had significantly fewer plaques in the aortic arch **(Figure 4A)**. Atherosclerosis is characterized by the accumulation of plaques and cholesterol 56, 57. Our study showed that treatment with GLSP significantly reduced plaque area throughout the aorta **(Figure 4B)**. The formation of vulnerable plaques is a risk factor for the pathogenesis of atherosclerosis. Vulnerable plaques are characterized by large necrotic cores and thin fibrous caps 58, 59. HE staining revealed that GLSP significantly reduced the area of necrotic cores and increased the thickness of fibrous caps (**Figure 4C**). Oil Red O staining of the aortic root also showed that GLSP intervention significantly reduced the plaque area in the aortic root **(Figure 4D)**. Additionally, under pathological conditions, VSMCs migrate to cover the plaques and form part of their composition 60. GLSP significantly increased the content of VSMCs within the plaques, contributing to plaque stability **(Figure 4E)**. Aging, as an independent risk factor for the development of atherosclerosis, typically leads senescent cells to secrete pro-inflammatory cytokines that accelerate the progression of atherosclerosis 4. By isolating primary peritoneal macrophages, we found that GLSP significantly reduced the expression of the pro-inflammatory factor TNF-α and increased the expression of the anti-inflammatory factor Arg1 **(Figure 4F-H)**. Additionally, we used flow cytometry to detect the number of pro-inflammatory monocytes in the peripheral blood of apoE^-/-^ mice. The results showed that GLSP significantly reduced the proportion of pro-inflammatory monocytes **(Figure 4I)**. Throughout the progression of atherosclerotic lesions, foam cells exhibiting senescence markers progressively accumulate beneath the endothelium 28. By extracting primary peritoneal macrophages and performing Oil Red O staining, we found that GLSP significantly reduced the number of foam cells **(Figure 4J)**. The formation of foam cells is closely linked to the macrophage-mediated uptake and efflux of cholesterol. During the development of atherosclerosis, macrophages regulate intracellular cholesterol homeostasis by scavenger receptor SRA-mediated cholesterol uptake and ATP-binding cassette transporter G1 (ABCG1)-mediated cholesterol efflux, thereby maintaining intracellular cholesterol homeostasis 61-63. Our study revealed that GLSP not only downregulated the expression of SRA but also upregulated the expression of ABCG1 within the plaques. Notably, similar findings were observed in primary peritoneal macrophages **(Figure S3A-E)**. These results suggested that GLSP exerted its protective effect by inhibiting foam cell formation through a dual mechanism: reducing cholesterol uptake and promoting cholesterol efflux, thereby mitigating the progression of atherosclerosis. Moreover, through comprehensive analysis via Western blot and PCR of senescence markers and inflammatory factors in primary peritoneal macrophages, it was evidenced that GLSP markedly decreased the expression of P16, P21, P53 and TNF-α, while concurrently enhancing the expression of Cycline1 and Arg1 **(Figure 4K-L)**. We also examined senescence markers within the plaques and found that GLSP significantly reduced P16 expression **(Figure 4M)**. It suggested that GLSP attenuated the aging process in atherosclerosis. In summary, these findings suggested that GLSP not only mitigated inflammatory responses but also attenuating foam cell formation, ultimately contributing to the amelioration of age-associated atherosclerosis.

### GLSP attenuated vascular calcification in aged mice as well as VD_3_ and nicotine-induced calcified aorta

**Figure 5:**
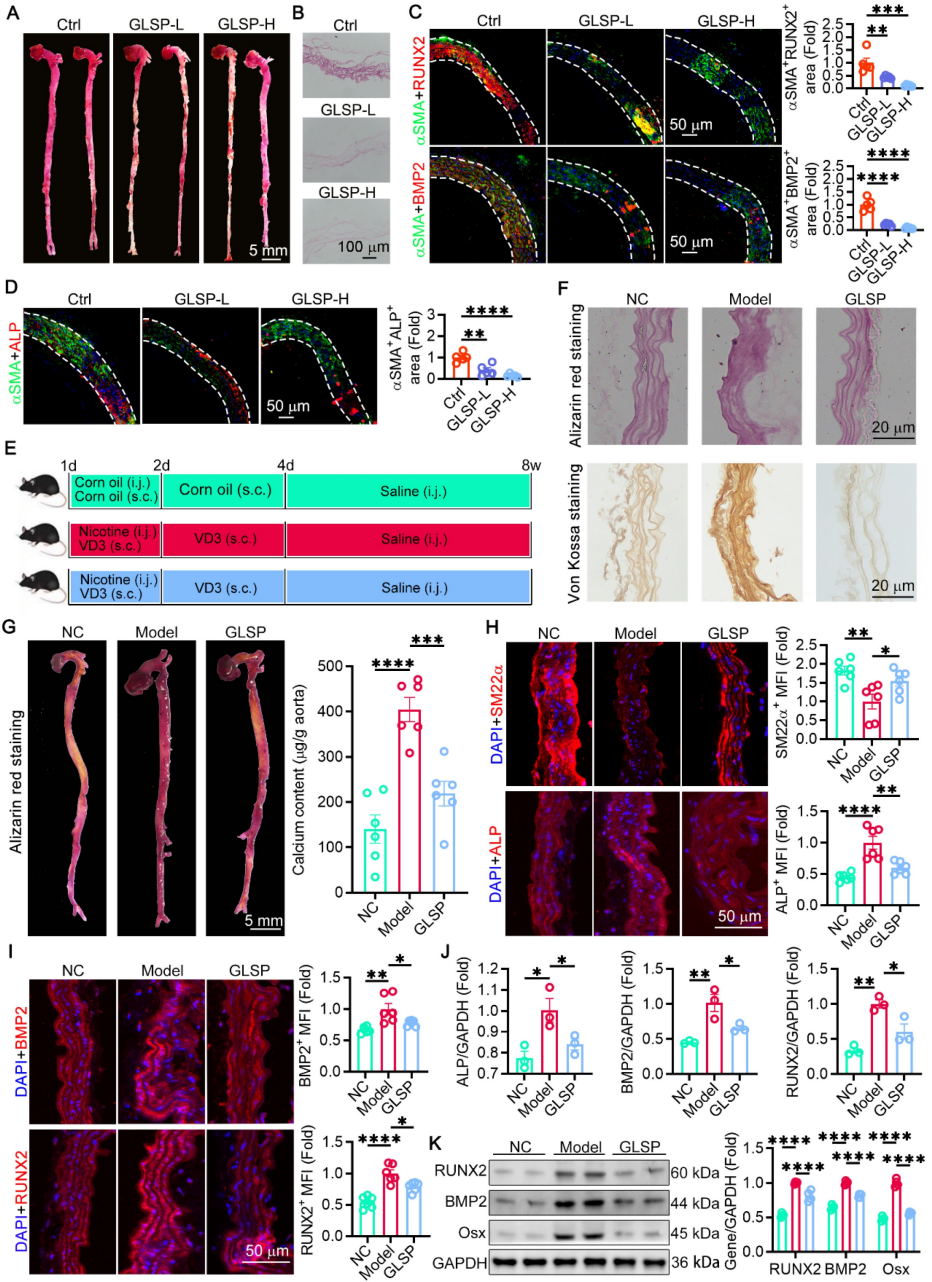
** GLSP alleviated vascular calcification. (A, B)** Alizarin red S staining was used to detect the degree of calcification in sections of the thoracic aorta and whole aorta (n = 5). **(C, D)** Immunofluorescence staining showed co-localization of αSMA with RUNX2, BMP2 and ALP (n = 5). **(E)** C57BL/6J mice were randomized into three groups and experimental protocols (n = 6). **(F)** Alizarin red S staining and Von Kossa staining of mouse thoracic aorta (n = 6). **(G)** Whole aorta alizarin red staining and quantification of total aortic calcium content (n = 6). **(H, I)** Immunofluorescence staining of molecules associated with calcification in mouse thoracic aorta (n = 6). **(J)** Expression levels of calcification-related molecules in mouse aorta detected by qRT-PCR (n = 3). **(K)** Quantification of RUNX2, BMP2 and Osx protein expression in the aorta of a calcified mouse model by western blot (n = 4). *P < 0.05, **P < 0.01, ***P < 0.001, ****P < 0.0001. All experiments were compared with the Ctrl or the Model group and error bars denote SEM.

The increase in vascular stiffness induced by vascular calcification and extracellular matrix remodeling is a hallmark of vascular aging 7. We observed that GLSP significantly reduced calcium deposition in the vascular wall in natural aging model mice **(Figure 5A-B)**. Simultaneously, following GLSP intervention, there was a notable reduction in the co-localization area of calcification-related genes RUNX2, BMP2, ALP and αSMA in aged aortas **(Figure 5C-D)**. To evaluate the effect of GLSP on medial calcification, we constructed the model of vascular calcification in C57BL/6J mice with subcutaneous injections of VD_3_ and then treated with GLSP for 8 weeks **(Figure 5E)**. Frozen sections of the mice thoracic aorta were subjected to Alizarin Red S and Von Kossa staining. The results demonstrated a pronounced intensification in coloration within the thoracic aorta of the vascular calcification mouse model, whereas the coloration became lighter following GLSP treatment **(Figure 5F)**. Additionally, Alizarin Red S staining was performed on the whole aorta of mice. The results showed that the aortic color significantly darkened in the mice model of vascular calcification, while it lightened following GLSP treatment, indicating that GLSP reduced calcium deposition in the aorta. Further analysis of the calcium content in the aorta revealed a significant increase in the mice model of vascular calcification, but a decrease with GLSP treatment **(Figure 5G)**. Osteogenic differentiation of VSMCs is the key to vascular calcification, and we found that the expression of SM22α in the thoracic aorta of mice was significantly decreased in the mice model of vascular calcification, and the expression of SM22α was significantly increased after GLSP treatment. Calcification-related molecules ALP, BMP2 and RUNX2 were significantly increased in the mice model of vascular calcification, but all were significantly decreased after GLSP treatment **(Figure 5H-I)**. Furthermore, the expression levels of ALP, BMP2 and RUNX2 were examined at the transcriptional level, and the results were consistent with the immunofluorescence staining results, which showed that the expression of ALP, BMP2 and RUNX2 were significantly down-regulated after treatment with GLSP **(Figure 5J)**. Subsequently, we assessed the expression levels of RUNX2, BMP2, and Osx proteins, revealing a marked reduction in their levels following the administration of GLSP **(Figure 5K)**. Finally, an *in vitro* model of calcification combined with senescence was established in VSMCs, where Alizarin Red staining and calcium content analysis demonstrated that the senescence inducer significantly promoted calcification. However, this effect was mitigated upon treatment with the GLSP components **(Figure S4A-B)**. In summary, these findings indicated that senescence promoted calcification, while GLSP and its active components exhibited an inhibitory effect on age-associated calcification.

### The components of GLSP mitigated VSMC senescence through reducing oxidative stress and improving mitochondrial function

**Figure 6:**
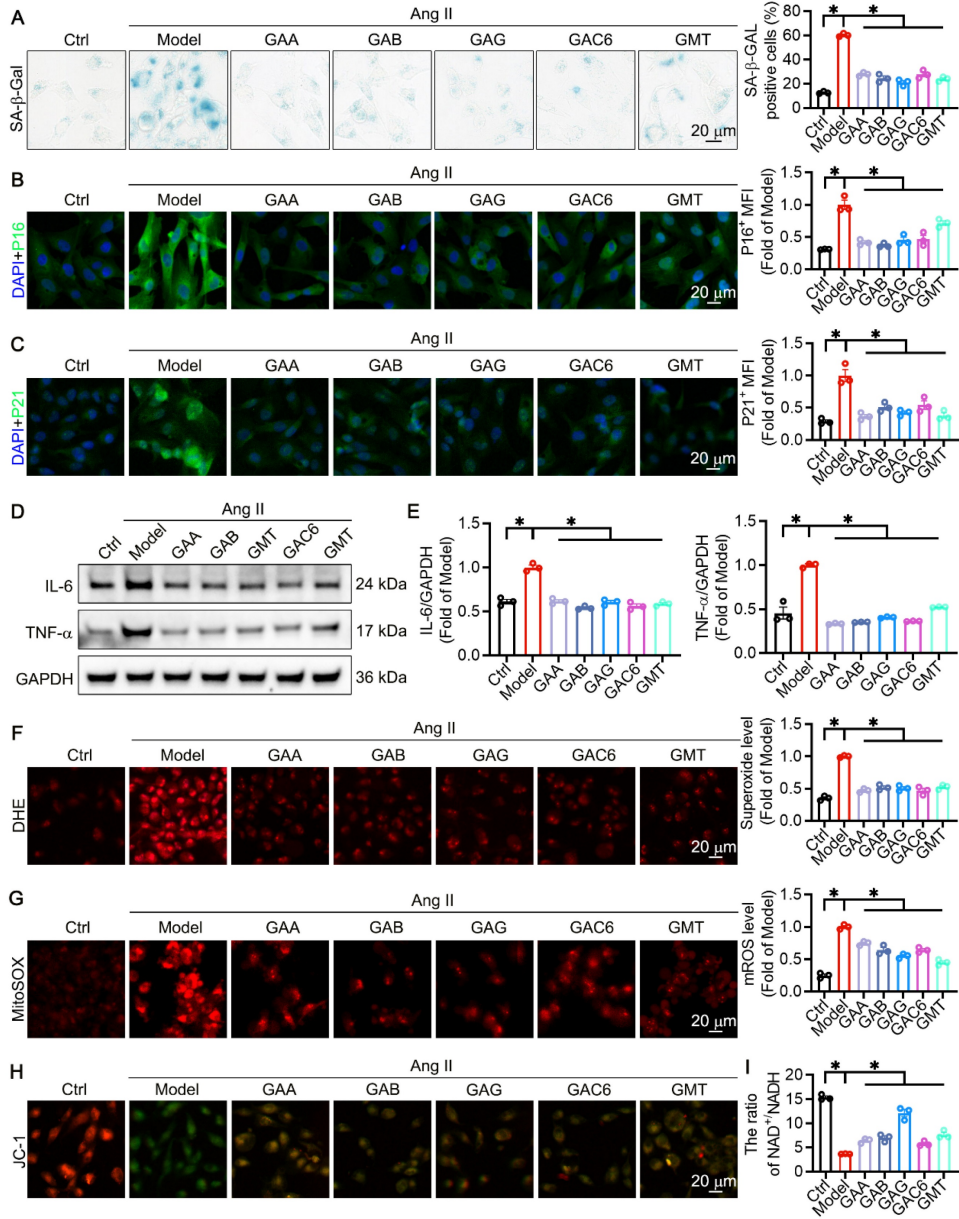
** The principal component of GLSP was capable of decelerating the aging process of VSMC *in vitro*. (A)** Senescence-associated beta-galactosidase staining and quantitative analysis were performed on VSMCs subjected to treatment with Ang II or one of GLSP component (n = 3). **(B, C)** Immunofluorescence staining was used to detect the expression of senescence markers p16 and p21 (n = 3). **(D, E)** Western blot results revealed the expression of the inflammatory factors IL-6 and TNF-α (n = 3). **(F, G)** Detection of total intracellular ROS and mitochondrial ROS production was carried out using the dihydroethidium probe and mitochondrial superoxide indicator probe (n = 3). **(H)** The JC-1 fluorescent probe was used to assess mitochondrial membrane potential (n = 3). **(I)** The ratio of NAD^+^ to NADH in VSMCs (n = 3). *P < 0.05. All experiments were compared with the Model group and error bars denote SEM.

In previous studies, we identified the primary triterpenoid active components of GLSP, including GAA, GAB, GAG, GAC6, and GMT, through HPLC, and determined their optimal concentrations for HASMCs using the CCK-8 assay 35. To assess the impact of the principal active components of GLSP on mitigating VSMC senescence, we employed Ang II, a well-characterized hormone related to vascular remolding and senescence 64, to induce VSMCs senescence. Subsequent SA-β-Gal staining demonstrated that active components of GLSP markedly diminished the quantity of senescent cells **(Figure 6A)**. The results of immunofluorescence staining demonstrated that the active components of GLSP decreased the expression of senescence markers p16 and p21 **(Figure 6B-C)**. Moreover, inflammatory responses are pivotal factors influencing the progression of aging 65, 66. We revealed that the active components of GLSP were effective in reducing inflammatory factors IL-6 and TNF-α **(Figure 6D-E)**. Mitochondria is integral to the regulation of the aging process. The decreasing efficacy of the respiratory chain in mitochondria resulted in elevated ROS production and a decrease in cellular ATP synthesis during aging 10, 67. We assessed the production of total ROS and mitochondrial ROS in VSMCs by employing DHE and MitoSOX probes, and discovered that the components of GLSP significantly reduced ROS levels **(Figure 6F-G)**. Additionally, there is a close relationship between mitochondrial membrane potential and ROS 68. Through JC-1 staining, it was observed that the active components of GLSP were capable of enhancing mitochondrial membrane potential **(Figure 6H)**. Furthermore, the ratio of NAD^+^ to NADH is intricately linked not only to redox capacity but also to mitochondrial metabolism. The active components of GLSP significantly enhanced the ratio of NAD^+^ to NADH **(Figure 6I)**. In summary, the active components of GLSP effectively modulated the redox status, sustain mitochondrial function and consequently retard the aging of VSMCs.

### GLSP improved vascular aging by regulating metabolism and promoting Sirt7 expression

**Figure 7:**
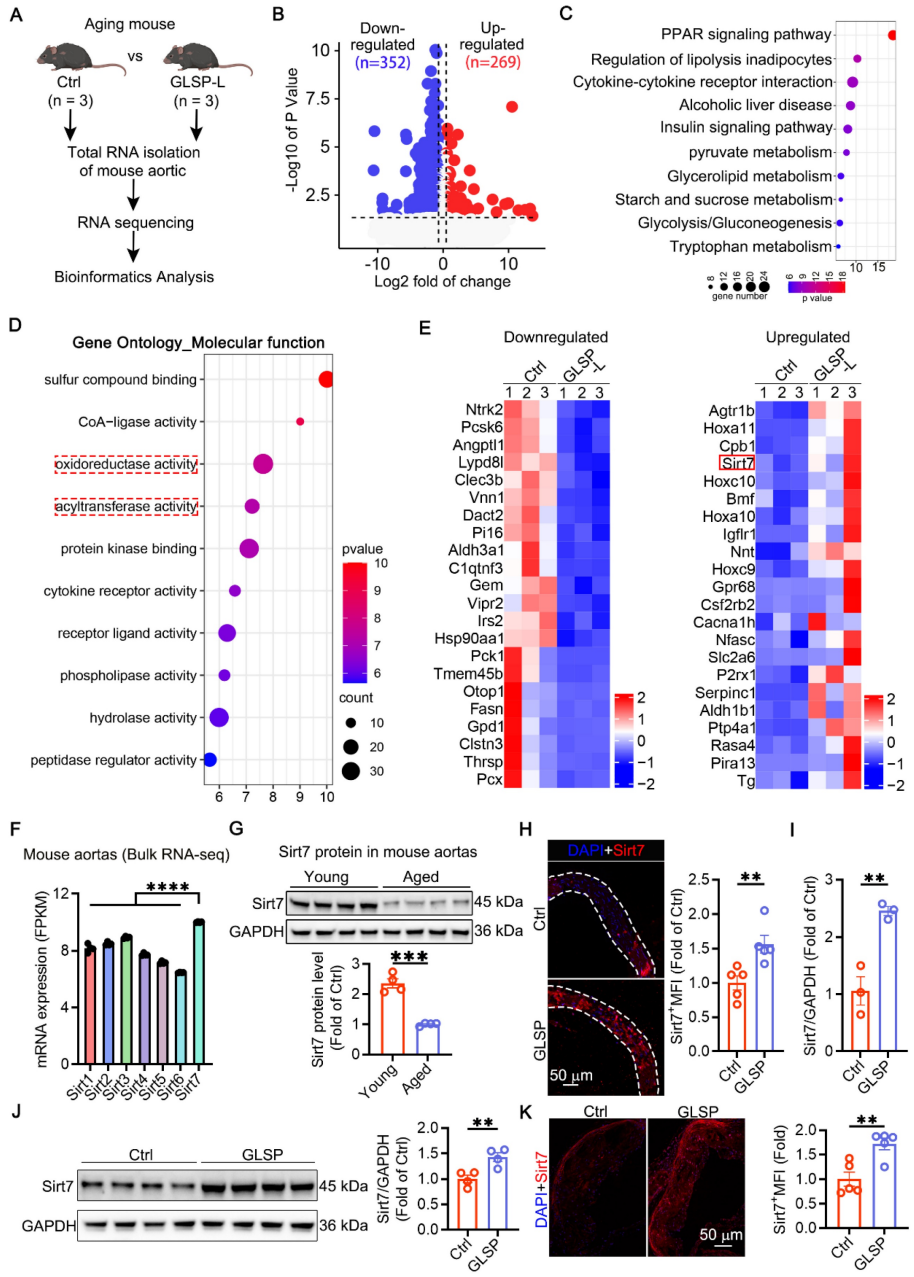
** GLSP mitigated vascular aging through regulating metabolic pathways. (A)** Study designed RNA sequencing to understand aortic transcriptome changes in GLSP-intervened aging mice (n = 3). **(B)** Volcano plot of RNA sequencing data showed 352 genes down-regulated and 269 genes up-regulated after GLSP intervention compared to the Ctrl group (n = 3). **(C)** GLSP treatment led to enrichment of differential genes in metabolic pathways. **(D)** GO molecular function enrichment analysis indicated that the differential genes are primarily involved in oxidative stress and acyltransferase activity. **(E)** The heatmap analysis visualized the expression patterns of upregulated and downregulated genes. **(F)** The basic expression levels of the Sirt family in the aorta of young mice in the GSE69187 database (n = 3). **(G)** Changes in Sirt7 protein expression between young and aged mice (n = 4). **(H)** Immunofluorescence staining was performed to detect changes in Sirt7 protein expression levels following GLSP treatment (n = 5). **(I)** qRT-PCR analysis of Sirt7 gene transcription levels (n = 3). **(J)** Western blot analysis of Sirt7 protein expression (n = 4). **(K)** Immunofluorescence detection of Sirt7 expression in plaques of apoE^-/-^ mice (n = 5). *P < 0.05, **P < 0.01, ***P < 0.001, ****P < 0.0001. All experiments were compared with the Ctrl group and error bars denote SEM.

To investigate the mechanism by which GLSP alleviates vascular aging, RNA sequencing was performed on aged mice from both the Ctrl and GLSP-treated groups** (Figure 7A)**. By applying a threshold of fold change > 1.5 and P < 0.05, we identified 269 genes that were upregulated and 352 genes that were downregulated compared to the Ctrl group **(Figure 7B)**. Kyoto Encyclopedia of Genes and Genomes (KEGG) and Gene Ontology (GO) molecular function enrichment analyses were conducted to further explore these differentially expressed genes. KEGG analysis revealed significant enrichment in metabolic pathways **(Figure 7C)**, while GO molecular function analysis indicated an enrichment of genes associated with oxidative stress following GLSP treatment **(Figure 7D)**. We then presented a heatmap to visually depict the upregulated and downregulated genes involved in the vascular aging process in response to GLSP treatment **(Figure 7E)**. Sirtuins (SIRTs) are conserved NAD^+^-dependent protein deacetylases that have beneficial effects against aging and metabolic diseases, and have been recognized as a potential effective target for cardiovascular disease 69-71. Sirt7 protein plays a crucial role in regulating energy metabolism, oxidative stress and the aging process, aligning with the findings from the enrichment analyses 72, 73. Transcriptomic data revealed that Sirt7 was the only sirtuin family member to show a significant increase in expression following GLSP treatment **(Figure S5A)**. Moreover, our evaluation of the mRNA levels of Sirt1-6 revealed no notable changes in their expression **(Figure S5B)**. By analyzing public databases, we observed that Sirt7 exhibited a high baseline expression level in the mice aorta** (Figure 7F)**. Notably, its expression in aged aortic tissue was lower than in young aortic tissue** (Figure 7G)**. GLSP treatment restored Sirt7 expression in aged aortic tissue and atherosclerotic plaques, consistent with the transcriptomic data **(Figure 7H-K)**. In summary, these findings suggested that GLSP may mitigate vascular aging by activaitng the Sirt7 signaling pathway.

### Sirt7 promoted deacetylation of Keap1 and Keap1-Nrf2 dissociation and by which GLSP mitigated vascular aging

**Figure 8:**
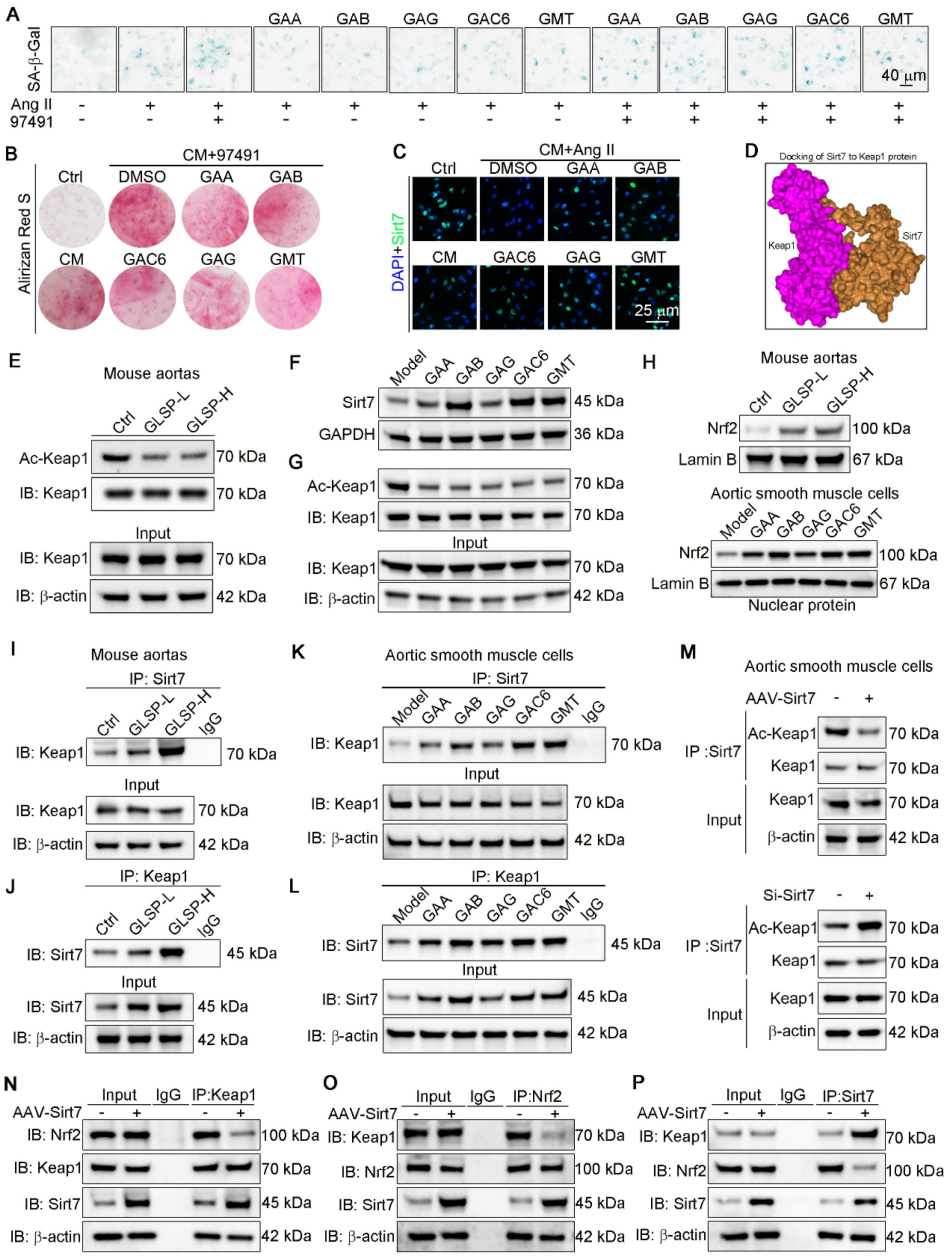
** GLSP activated Sirt7-Nrf2 pathway by deacetylation of Keap1. (A)** Representative images of β-galactosidase staining after treatment with the Sirt7 inhibitor 97491 (5 μm) and the GLSP monomer component (n = 3). **(B)** Representative images of Alizarin Red staining after treatment with a Sirt7 inhibitor 97491 (5 μm) and the GLSP monomer component (n = 3). **(C)** Immunofluorescence staining showed the expression level of Sirt7 under senescence and calcification culture conditions (n = 3). **(D)** Molecular docking with the HDOCK server predicted the binding potential between Sirt7 and Keap1 (http://hdock.phys.hust.edu.cn). **(E)** Aortic lysates were immunoprecipitated using Keap1 antibody, followed by blotting with an anti-acetylation antibody (n = 5). **(F)** Immunoblotting was used to detect the expression levels of Sirt7 *in vitro* in VSMCs (n = 3). **(G)** Cell lysates were immunoprecipitated using Keap1 antibody, followed by blotting with an anti-acetylation antibody (n = 3). **(H)** Immunoblotting was used to detect the expression of nuclear Nrf2 in the aorta and VSMCs (n = 3). **(I-L)** The effects of GLSP and its components on the interaction between Sirt7 and Keap1 proteins in the aorta and VSMCs were evaluated through co-IP and immunoblotting. **(M)** Co-IP and immunoblotting were used to evaluate the effect of overexpressing or silencing Sirt7 on the acetylation of Keap1 (n = 3). **(N-P)** After overexpression of Sirt7 in VSMCs by infected with AAV-Sirt7, the interaction between Keap1 and Nrf2 was determined by co-IP. *P < 0.05, **P < 0.01, ***P < 0.001, ****P < 0.0001. All experiments were compared with the Ctrl group or the Model group and error bars denote SEM.

Cellular senescence has been implicated in the pathogenesis of vascular calcification 74. Sirt7 plays a protective role against calcification and senescence of VSMCs 75. To determine the role of Sirt7 in inhibiting vascular aging, we used a Sirt7 inhibitor to suppress the activity of the Sirt7 deacetylase, as reported in the literature 76. Subsequently, we performed β-galactosidase staining, which showed a significant increase in the number of senescent cells following Ang II stimulation. Treatment with the Sirt7 inhibitor further exacerbated cell senescence and abolished the anti-senescence effects of GLSP components **(Figure 8A)**. In addition, Sirt7 inhibition promoted the calcification of senescent VSMCs and abolished the inhibitory effect of GLSP on VSMCs calcification **(Figure 8B)**. These results suggested that the GLSP components mediate their anti-aging properties through activating Sirt7. Importantly, we observed that the active components of GLSP significantly enhanced the expression of Sirt7 under the combined conditions of calcification and cell senescence **(Figure 8C)**. Keap1/Nrf2 signaling pathway plays important role in aging and aging-associated diseases 77-80. More importantly, the acetylation status of Keap1 determined its function 81. Since the Sirt7 is a NAD^+^-dependent deacetylase, we hypothesized that Sirt7 regulated Keap1 through influencing its acetylation status. Subsequently, the interaction between Sirt7 and Keap1 was predicted by molecular docking experiments **(Figure 8D)**. Furthermore, we found that GLSP reduced the Keap1 acetylation *in vivo* and the components of GLSP reduced the Keap1 acetylation *in vitro* **(Figure 8E, G)**, which may be attributed to the GLSP-induced Sirt7 expression **(Figure 7E and 8F)**. Activation of Nrf2 relies on the liberation from Keap1-mediated repression 82-84. Noticeably, we observed that downregulation of Keap1 acetylation promoted Keap1-Nrf2 dissociation**,** which promoted Nrf2 nuclear translocation and activation **(Figure 8H)**. Subsequently, we confirmed that Sirt7 physically interacted with Keap1 in co-IP assays **(Figure 8I-L)**. To further determine the interaction between Sirt7 and Keap1, we overexpressed or knock down the Sirt7 in the VSMCs, and then assessed the acetylation level of Keap1. The result showed that Keap1 acetylation level decreased in AAV-Sirt7 VSMCs compared with WT VSMCs **(Figure 8M, up panel)**. Conversely, the Keap1 acetylation level increased in Sirt7 knockdown VSMCs compared with WT VSMCs **(Figure 8M, down panel)**. In addition, the co-IP experiment showed that overexpression of Sirt7 weakened the interaction between Keap1 and Nrf2 **(Figure 8N-P)**. Taken together, these results suggested that Sirt7 deacetylated Keap1 and the deacetylation of Keap1 promoted Keap1-Nrf2 dissociation and the following Nrf2 activation.

## Discussion

Age-related pathological alterations of the vasculature, such as atherosclerosis, have a critical role in morbidity and mortality of older adults 1, 85. Our previous study showed that GLSP has the potential to ameliorate vascular aging and cardiovascular disease, but little is known about its pharmacological effects. In this study, GLSP was shown to improve vascular aging phenotypes in aged mice, including reducing vascular wall thickening, reducing collagen deposition, decreasing vascular permeability, cell cycle arrest, inflammation, oxidative stress, mitochondrial dysfunction and mitophagy dysregulation. In addition, GLSP alleviated advanced atherosclerosis and vascular calcification in aged mice. Mechanistically, GLSP enhanced Sirt7 expression. Sirt7 deacetylated Keap1 and promoted Keap1-Nrf2 dissociation and the following Nrf2 activation, which facilitated the upregulation of antioxidant factors and thereby alleviating vascular aging. In summary, these results indicated that GLSP mitigated vascular aging, atherosclerosis and vascular calcification through activating the Sirt7-Nrf2 axis.

The essence of vascular aging lies in the senescence of endothelial cells and VSMCs, with one of the key characteristics of cellular senescence being cell cycle arrest 86, 87. Senescent cells typically remain in the G1, S, or G2 phase of the cell division cycle, which is induced by a series of endogenous or exogenous stresses, such as ROS, telomere attrition and DNA damage. These stresses lead to increased expression of P53, P21 or P16 proteins, which in turn reduce the expression of cyclin-dependent kinases 17, 88, 89. This study observed that GLSP reduced the expression of P53, P21 and P16 proteins and reduced the phosphorylation levels of H2AX and Chk1, indicating its ability to intervene in the DNA damage response. Oxidative stress and inflammation drive the onset and progression of vascular aging 90. ROS primarily originate from mitochondria and is mainly attributed to the dysfunction of the mitochondrial electron transport chain 47. Additionally, defect of Nrf2-mediated antioxidant systems further triggered the DNA damage and accelerated cellular senescence 9. Therefore, targeting the balance of redox reactions and reducing oxidative stress may be potential strategies for alleviating vascular aging. Our results indicated that GLSP could reduce inflammation and ROS levels in the aorta, as well as decrease oxidative damage to proteins, lipids and DNA. Damaged mitochondria continuously produces ROS and release pro-apoptotic signals that induce apoptosis in healthy cells 91, indicating that the clearance of damaged mitochondria is crucial for mitochondrial health. Mitophagy refers to the process by which damaged or dysfunctional mitochondria are selectively degraded through the autophagy pathway 92. Clinical studies have shown that impaired mitophagy negatively affects cellular health and contributes to age-related chronic diseases 92. Mitophagy is primarily induced through the ubiquitin-dependent PINK1-Parkin pathway and the ubiquitin-independent pathway, where LC3 directly binds with autophagy proteins to initiate autophagy 93. Importantly, enhancing mitophagy in aged, hyperlipidemic mice prevented the increase in aortic IL-6 and Parkin, attenuated mitochondrial dysfunction and reduced atherogenesis 85. Our study indicated that GLSP reduced the inflammation and alleviated mitophagy dysfunction, which contributed to slowing down vascular aging and aging-associated atherosclerosis.

Atherosclerosis and vascular calcification are characteristic of vascular aging 22. As age increases, the decline in endothelial cell function due to inflammation and oxidative stress leads to increased vascular wall permeability, allowing more lipids to enter the vascular wall and form unstable plaques 57. Additionally, VSMCs undergo a transition from a contractile phenotype to a synthetic phenotype, which contribute to plaque formation 54, 94. Furthermore, the breakdown of elastic fibers and vascular matrix remodeling lead to structural changes in the vascular wall, causing the vascular to become stiff and increasing plaque instability. Senescent cells also secrete pro-inflammatory cytokines that attract monocytes and other inflammatory cells to the vascular wall, accelerating the progression of atherosclerosis 95. GLSP reduced the plaques and necrotic cores in mice with advanced atherosclerosis, lowered the expression levels of senescence markers and decreased the number of inflammatory cells in peripheral blood, suggesting that slowing down vascular aging may alleviate atherosclerosis. Vascular calcification is caused by the deposition of hydroxyapatite in the vascular wall, which is mainly divided into intimal arterial calcification and medial arterial calcification 96. Medial arterial calcification results in vascular stiffness and increases pulse pressure, which elevates cardiac load and ultimately leads to left ventricular hypertrophy and diastolic dysfunction 97. In addition, vascular calcification is an independent risk factor for end-stage renal disease 98. Intimal arterial calcification occurs mainly into the atherosclerotic plaques, leading to plaque instability 99. The underlying mechanism of vascular calcification is mainly related to the transformation of VSMCs to osteoblast-like cells. Oxidative stress and inflammation induce phenotypic transformation of VSMCs from a contractile phenotype to a secretory phenotype and differentiation towards osteogenesis 100. A recent study demonstrates a significant increase in calcium deposition in the aortic valves of aging mice, highlighting aging as an important factor contributing to vascular calcification 101. In aged mice, GLSP reduced the calcified area and decreased the expression of pro-calcification genes RUNX2, BMP2 and ALP in the vascular. Similar results were observed in mice with vascular calcification induced by VD_3_ and nicotine. These findings suggested that GLSP can improve calcification during the aging process. Furthermore, age-related abdominal aortic aneurysm (AAA) is characterized by the infiltration of inflammatory cells into the vascular wall and the degradation of elastic fibers 102. Additionally, aging leads to abnormal vascular cell function, increased oxidative free radicals and vascular inflammation, which affect the structure and function of the elastic vascular wall, thereby promoting hypertension 103. GLSP not only possesses antioxidant and anti-inflammatory properties and improves mitochondrial function but also improves vascular wall remodeling, suggesting that GLSP has the potential to attenuate AAA and hypertension.

Nrf2, a pivotal transcription factor in maintaining cellular redox homeostasis, exerts a profound influence in aging, atherosclerosis, and vascular calcification 79, 104-106. Its activation drives the expression of key downstream antioxidant genes, such as NQO1 and HMOX1, thereby conferring cell against oxidative damage 107. In addition to its antioxidative roles, Nrf2 activation mitigates inflammation, positioning it as a critical factor in delaying aging and combating vascular diseases 108. Notably, Nrf2 activity is tightly regulated by the Keap1 protein. Keap1 maintains Nrf2 in an inactive state through a continuous process of ubiquitination, marking Nrf2 for degradation by the CUL3 E3 ubiquitin ligase complex 109. In its basal state, Keap1 forms a complex with Nrf2, preventing its nuclear translocation and activation. Hence, disrupting the Keap1-Nrf2 interaction promotes Nrf2's entry into the nucleus, enabling its transcriptional activity. Keap1 undergoes diverse post-translational modifications, including ubiquitination, acetylation, alkylation, and glycosylation, which influence its function and interaction with Nrf2 81, 110. Despite that the role of Keap1 ubiquitination in releasing Nrf2 have been highlighted, the regulatory impact of Keap1 acetylation remains poorly understood 111. Sirtuins (SIRTs), a family of NAD^+^-dependent deacetylases, counteract aging and metabolic disorders, with emerging roles in cardiovascular health 112. Sirt1 maintains endothelial function by reducing oxidative stress and inflammation, thereby suppressing atherosclerosis 86, while also regulating VSMC dynamics to improve vasodilation 113. Sirt2 mitigates cardiac aging and oxidative damage 114, 115, whereas mitochondrial-localized Sirt3-5 govern energy metabolism 116. Chromatin-associated Sirt6 attenuates cardiovascular inflammation via NF-κB inhibition 117. Notably, Sirt7 emerges as a critical lifespan regulator: its decline correlates with stem cell aging 118, while its deficiency impairs DNA repair and accelerates genomic instability 119 Sirt7 activates the GABPα/β complex to enhance mitochondrial function and delay aging 120. Crucially, our study identified Sirt7 as the primary mediator of GLSP's anti-aging effects in vascular systems, with no significant changes observed in Sirt1-6 expression. We found that GLSP and its active components reduced Keap1 acetylation via Sirt7 upregulation, disrupting Keap1-Nrf2 binding to promote Nrf2 release, nuclear translocation and antioxidant gene activation. These results highlight Sirt7's central role in regulating Keap1/Nrf2 signaling and its therapeutic potential for aging-related vascular pathologies like atherosclerosis and vascular calcification. Under certain specific conditions, Nrf2 exhibits Keap1-independent activation via post-translational modifications, including phosphorylation, deacetylation, and deubiquitination 121-123. Our study identified Sirt7 as a key modulator of Keap1 acetylation and Nrf2 activation, suggesting its therapeutic potential for vascular aging pathologies, such as atherosclerosis and vascular calcification.

A key hallmark of the aging process is the impaired ability of senescent cells to respond to molecular stress. Vascular aging involves Nrf2 dysfunction that amplifies oxidative stress and enhances ROS sensitivity in endothelial/smooth muscle cells 124, 125. We found that Sirt7/Keap1/Nrf2 pathway enhances cellular stress resistance by driving Nrf2 nuclear translocation and antioxidant gene expression. Anti-aging mechanisms include AMPK, which extends lifespan through catabolic activation and ATP production 126, and the mTOR/AKT-PKC-SGK axis that accelerates aging via FOXO1 inhibition and pro-inflammatory NF-κB activation 127. AMPK/mTOR primarily respond to nutrient status. During aging, the efficiency of mitochondrial energy production significantly decreases, leading to an inability of cells to maintain normal metabolic activities and repair capabilities, resulting in the decline of tissue and organ function 128. Additionally, dysfunctional mitochondria release damaged mitochondrial DNA, triggering apoptosis and inflammatory responses 129. Nrf2 acts as a regulator of mitochondrial function 130. Our study demonstrated that GLSP can improve mitochondrial function and enhance mitophagy in aging aortas, which may be attributed to the Nrf2 activation. Collectively, this study indicated that there may be crosstalk between AMPK/mTOR and Sirt7/Keap1/Nrf2 pathways. Investigating crosstalk between these pathways may advance anti-aging strategies.

In conclusion, GLSP alleviated vascular aging and age-associated atherosclerosis and calcification by activating Sirt7. Mechanistically, GLSP enhanced Sirt7-mediated deacetylation of Keap1, by which GLSP promoted Keap1-Nrf2 dissociation and the following Nrf2 activation. This study suggested that Sirt7 may be a target for treating vascular aging, and GLSP may be a new strategy for treating vascular aging, and aging-associated atherosclerosis and vascular calcification.

## Methods

### Reagents

Antibodies for P16, (CAT# 10883-1-AP); P21, (CAT# 67362-1-Ig); P53, (CAT# 60283-2-Ig); TNF-α, (CAT# 60291-1-Ig); MMP3, (CAT# 66338-1-Ig); MMP13, (CAT# 18165-1-AP); TOM20, (CAT# 66777-1-Ig); Arg1, (CAT# 66129-1-Ig); αSMA, (CAT# 67735-1-Ig); ABCG1, (CAT# 13578-1-AP); Sirt7, (CAT# 12994-1-AP); HMOX1, (CAT# 66743-1-Ig); NQO1, (CAT# 67240-1-Ig); LC3, (CAT# 14600-1-AP); P62, (CAT# 31403-1-AP); PINK1, (CAT# 23274-1-AP); BMP2, (CAT# 66383-1-Ig); IL-6, (CAT# 26404-1-AP); Keap1, (CAT# 60027-1-Ig); Nrf2, (CAT# 16396-1-AP) were purchased from Proteintech Group, Inc. Antibodies for IL-1β, (CAT# sc-515598); ICAM-1, (CAT# sc-107); VCAM-1, (CAT# sc-13160); CD68, (CAT# sc-20060); SR-A, (CAT# sc-20445); RUNX2, (CAT# sc-390351); ALP, (CAT# sc-365765); Osx, (CAT# sc-393060) were purchased from Santa Cruz Biotechnology, Inc. Antibodies for 3-nitrotyrosine, (CAT# bs-8551R); 4-hydroxynonenal, (CAT# bs-6313R) were purchased from BIOSS. Antibodies for 8-oxoguanine, (CAT# MAB3560) was purchased from Sigma-Aldrich. Antibodies for PH3, (CAT# ab177218); γH2AX, (CAT# ab81299); p-Chk1, (CAT# ab278717) were purchased from abcam. Flow Antibodies for CD11b, (CAT# 101263); CCR2, (CAT# 150627); Ly6C, (CAT# 128017) were purchased from BioLegend, Inc. (San Diego, CA). The antibody dilution ratios, species origin, and other information were provided in Table S2.

Senescence β-Galactosidase Staining Kit, (CAT# C0602); Mitochondrial Membrane Potential Detection Kit (JC-1), (CAT# C2006); NAD^+^/NADH Assay Kit with WST-8, (CAT# S0175) were purchased from Beyotime Biotechnology. Modified Sirius Red Stain Kit, (CAT# G1472); Collagen Fiber and Elastic Fiber Staining Kit, (CAT# G1597); Alizarin Red S solution, (CAT# G3280) were purchased from Solarbio Science & Technology Co., Ltd (Beijing, China). Dihydroethidium, (CAT# D11347); MitoSOX, (CAT# M36008); DMEM/F-12 medium, (CAT# 11330032) were purchased from Thermo Fisher Scientific. Malondialdehyde (MDA) Colorimetric Assay Kit (TBA Method), (CAT# E-BC-K025-M); Catalase (CAT) Activity Assay Kit, (CAT# E-BC-K031-M); Mouse TNF-α ELISA Kit, (CAT# E-EL-M3063); Mouse IL-1β ELISA Kit, (CAT# E-EL-M0037) were purchased from Elabscience. Oil Red O Powder, (Cat# 1320-06-5) and Calcium Colorimetric Assay Kit, (Cat# MAK022) were purchased from Sigma-Aldrich Inc. (Missouri, USA). MolPure® Cell/Tissue Total RNA Kit, (Cat# 19221ES50) was purchased from Yeasen Biotechnology Co., Ltd (Shanghai, China). HiScript II Reverse Transcriptase Kit, (Cat# R223) was purchased from Vazyme Biotech Co., Ltd. (Nanjing, China). Ultra SYBR Mixture, (Cat# CW2601H) was purchased from Cowin Bio. (Beijing, China). Fetal bovine serum, (CAT# C04001-500) was purchased from VivaCell Biosciences Ltd (Shanghai, China). Penicillin-streptomycin, (CAT# G4003-100 mL) was purchased from Servicebio Technology Co., Ltd., (Wuhan, China). The *Ganoderma lucidum* spore powder, (CAT# 20231202Y) was provided by Zhejiang Shouxiangu Pharmaceutical Co., Ltd., (Zhejiang China). GAA, (CAT# 81907-62-2); GAB, (CAT# 81907-61-1); GAG, (CAT# 98665-22-6); GAC6, (CAT# 105742-76-5); GMT, (CAT# 106518-63-2) were purchased from Chengdu Push Bio-technology Co., Ltd., (Chengdu China). Sirt7 inhibitor 97491, (CAT# 1807758-81-1) was purchased from MedChemexpress LLC (Monmouth Junnction, NJ, USA).

### Experimental animals in aging studies

Sixteen-month-old aging mice were purchased from Jiangsu Aniphe Biolaboratory Inc and housed in Tianjin Yishengyuan Biotechnology Co Ltd. Sixteen-month-old C57BL/6J male mice were randomly divided into three groups, control group (Ctrl), low-dose group of *Ganoderma lucidum* spore powder (GLSP-L) and high-dose group of *Ganoderma lucidum* spore powder (GLSP-H). In clinical practice, a normal 70 kg adult is recommended to consume 2 g of GLSP. According to the human-mouse body surface area conversion formula: mouse dose = 12.3 × adult dose 131, the equivalent dose for mice can be calculated as 351 mg/kg body weight/day. Considering that each mouse weighs 20 g, it would require a daily intake of 7 mg of GLSP. With a daily food consumption of 5 g, the drug content in the food would be 7 mg/5 g, which is 1400 mg/kg food weight/day. In this study, the low-dose group of GLSP was treated by gavage at a dose of 351 mg/kg body weight/day (equivalent to 1400 mg/kg food weight/day), while the high-dose group of GLSP was treated by gavage at a dose of 702 mg/kg body weight/day (equivalent to 2800 mg/kg food weight/day). During the administration period, the weight of the mice was recorded weekly. After 6 months, mouse serum, aorta, heart, liver, spleen, kidney and other tissues were collected for further research.

ApoE^-/-^ mice were purchased from Changzhou Cavens Experimental Animals Co., Ltd. and bred at Tianjin Yishengyuan Biotechnology Co., Ltd. Two-month-old male apoE^-/-^ mice were randomly assigned to two groups: the Ctrl group and the GLSP treatment group. The Ctrl group was fed a high-fat diet (HFD, 21% fat, 0.15% cholesterol, MD12015HL, Medicience Ltd.) to establish an atherosclerosis model. The GLSP treatment group received the same high-fat diet in addition to oral administration of GLSP at a dose of 351 mg/kg body weight/day (equivalent to 1400 mg/kg food weight/day). After 9 months of continuous high-fat feeding and gavage treatment, the mice were sacrificed for further research.

Wild-type C57BL/6J mice were purchased from Beijing Vital River Laboratory Animal Technology Co., Ltd. They were used as a model for medial calcification by gavage of nicotine and subcutaneous injection of VD_3_. Specifically, mice were randomly divided into three groups, mice in the model and treatment groups were gavage with nicotine (20 mg/kg) on the first day, VD_3_ (5.5×10^5^ IU/kg) was injected subcutaneously from the first day to the fourth day. Subsequently, the mice in the treatment group were administered GLSP by gavage at a dose of 351 mg/kg body weight/day (equivalent to 1400 mg/kg food weight/day) for 8 weeks.

All mice were adaptively fed for 7 days prior to the start of the experiment and placed under standard conditions with 12 h of light/dark circulation. Mice were free to access water and standard food. All animal care and *in vivo* experimental protocols conformed to the Guide for the Care and Use of Laboratory Animals published by the NIH (NIH publication no. 85-23, revised 1996). The protocols were approved by the First Teaching Hospital of Tianjin University of Traditional Chinese Medicine. This study was approved by the Animal Ethics Committee of Yishengyuan Gene Technology (Tianjin) Co., Ltd. (approval No.YSY-DWLL-2022437).

### Evans blue staining

At the end of the gavage period, a 0.5% Evans blue sterile solution was prepared in PBS. Then, 200 μL of the solution was injected via the tail vein into the mice, and the mice were kept stationary for 30 min. After euthanizing the mice, the aortas were quickly dissected and representative images were captured using a stereomicroscope (Olympus, SZX10-3121). Additionally, the aortas were weighed and 500 μL of formamide was added to each sample tube. The samples were then incubated in a 55 °C water bath for 24 h. The absorbance at 610 nm was measured and the amount of Evans blue extravasation per mg of tissue was calculated.

### Senescence-associated-β-galactosidase (SA-β -gal) staining

According to the manufacturer's instructions, a Senescence β-Galactosidase Staining Kit was used to assess the number of senescent cells. Cultured cells and freshly dissected aorta samples were rinsed three times in PBS buffer, then fixed at room temperature for 16 min using the β-galactosidase staining fixative. After washing three times with PBS buffer, the cells or aortic samples were placed in staining solution and incubated at 37 °C for 24 h. Images were captured using an optical microscope (Olympus, BX43) or camera and then analyzed with ImageJ software. The degree of senescence (blue-green) was evaluated based on a graded scoring system.

### Sirius Red staining and Verhoeff's Van Gieson (EVG) staining

The sections were submerged in phosphate-buffered saline (PBS) for 10 min to thoroughly remove the embedding medium. For Sirius red staining, the sections were immersed in Sirius red staining solution for 30 min. Subsequently, ethanol was used for gradient dehydration, xylene was used for transparency, and the slices were sealed with neutral resin. Images were captured using an upright fluorescence microscope (Leica, DM6000B), and collagen content was quantified using ImageJ software. For EVG staining, following the kit instructions, The VG stain was applied to the thoracic aorta for 10 min according to the kit instructions, rinsed with distilled water until clear, then stained with the working stain for 5 min and differentiated for 5-10 s. Finally, ethanol was used for gradient dehydration, xylene was used for transparency, and the slices were sealed with neutral resin. Images were captured using a light microscope (Olympus, BX43) and the number of elastic fiber fractures were quantified using ImageJ software.

### Immunofluorescence staining

Frozen sections of the thoracic aorta were immersed in PBS to remove the O.C.T. embedding medium. Tissues or cells were then permeabilized using Enhanced Immunostaining Permeabilization Buffer (Beyotime Biotechnology, CAT# P0097) for 5 min. After permeabilization, sections were washed three times with PBS for 5 min each, followed by blocking with 2% BSA for 1 h. The corresponding primary antibody was applied and incubated overnight at 4 °C. Following primary antibody incubation, it was gently blotted off, and the tissues or cells were washed three times with PBS for 5 min each. The secondary antibody was applied and incubated at 37 °C for 1 h. Finally, samples were mounted using an anti-fluorescence quenching mounting solution containing DAPI. Fluorescent images were captured with a fluorescence microscope (Leica, DM6000B), and mean fluorescence intensity or the area of positive regions was quantified using ImageJ software.

### Dihydroethidium staining and MitoSOX Red staining

Dihydroethidium (DHE) and MitoSOX staining were performed to monitor the levels of total superoxide and mROS. In brief, frozen sections and HASMCs were stained with 5 μm DHE or 5 μm MitoSOX for 30 min at 37 °C in a constant temperature incubator. Images were taken by Positive fluorescence microscope (Leica, DM6000B). Mean fluorescence intensity was counted with ImageJ software.

### Oil Red O staining

A stock solution of Oil Red O was prepared by dissolving 5 g of powder in 1 L of isopropanol. Before use, the stock solution was diluted with distilled water at a 2:3 ratio and filtered to create the working solution. For staining the *en face* aorta and aortic root sections, tissues were stained with the working solution for 1 h, rinsed with water until clear, and imaged using a stereomicroscope (OPTEC, SZ680) and a light microscope (Olympus, BX43). For primary peritoneal macrophages, cells were fixed with 4% paraformaldehyde for 30 min, stained with the working solution for 1 h, rinsed with water until clear, and counterstained with hematoxylin to visualize nuclei. Images were acquired using a light microscope. Atherosclerotic lesion areas and foam cell counts were quantified and analyzed using ImageJ software.

### Alizarin red S staining and Von Kossa staining

Alizarin red S staining of the whole aorta, thoracic aorta and HASMCs were used to detect calcium deposits. Briefly, the tissues and cells were submerged in alizarin red S stain for 10 min, then differentiated with McGee-Russell differentiation solution for 5 s, the whole aorta, the frozen sections of thoracic aorta and HASMCs were washed with PBS until the solution was colorless, the whole aorta and HASMCs were photographed by a stereomicroscope (OFNTC, SZ680), and the frozen sections thoracic aorta were photographed by a light microscope (Olympus, BX43). In addition, frozen sections of the thoracic aorta were stained by Von Kossa for the evaluation of calcium deposits in the vascular. Briefly, tissues were submerged in Von Kossa silver solution and irradiated under UV light for 15 min, then sections were submerged in seaborne solution for 2 min, and, the frozen sections thoracic aorta were photographed by a light microscope (Olympus, BX43).

### Quantitative real-time polymerase chain reaction

To test the mRNA levels of the targeted genes, total RNA was extracted from aorta and cells using an RNA extraction kit (Yeasen Biotechnology, CAT#19231ES50). Total RNA was reverse-transcribed to cDNA synthesis using a First Strand cDNA Synthesis Kit (Vazyme Biotech, CAT# R222-01). Quantitative real-time PCR (qRT-PCR) was performed to detect the mRNA levels of the target genes using AceQ qPCR SYBR Green Master Mix (Cowin, CAT#CW2601H). The thermocycling conditions used for qRT-PCR amplification were as follows: 5 min at 95 °C for pre-denaturation and 40 consecutive cycles of amplification (10 s at 95 °C for denaturation, 30 s at 60 °C for annealing and extension). GAPDH was used to normalize mRNA levels using the 2^^-ΔΔCt^ method. The primers were listed in Table S1.

### RNA sequencing

After removal of excess fat and blood from mouse aortas, they were immediately placed in 1.5 mL EP tubes and snap-frozen in liquid nitrogen. The samples were then buried in dry ice and sent to GENE DENOVO for testing. The images in this article were downloaded from the biorender website (https://www.biorender.com). GO and KEGG enrichment analyses were performed on the microbial bioinformatics website (http://www.bioinformatics.com.cn).

### Cell culture

In this experiment, HASMCs were used for subsequent experiments and maintained in DMEM/F12 medium (Gibco, CAT# 11330032), 10% fetal bovine serum (VivaCell Biosciences, CAT# C04001-500), and 1% penicillin-streptomycin mixture (Servicebio Technology, CAT# G4003-100 mL). The cells were cultured at 37 °C in a constant temperature incubator containing 5% carbon dioxide.

### Cellular senescence model and cellular calcification model

To induce senescence in HASMCs, cells were seeded in a plate and treated with Ang II (0.6 μM, Sigma, CAT# A6778) for 5 days to induce senescence, while simultaneously administering the active monomeric components of GLSP, namely GAA (10 μM), GAB (10 μM), GAG (10 μM), GAC6 (10 μM) and GMT (10 μM). To inhibit the activity of Sirt7 deacetylase *in vitro*, Sirt7 inhibitor 97491 (5 μM, MCE, CAT# HY-135899) was used for the first two days. During this period, the culture medium was replaced every day to prevent the loss of efficacy of Ang II and the monomeric active components.

HASMCs were induced calcification by culture in complete DMEM/F12 medium (1:1) containing 5 mM Pi (mixture of NaH_2_PO_4_ and Na_2_HPO_4_, ratio 1:2, pH7.4) and 50 μg/mL ascorbic acid for 5 days. To inhibit the activity of Sirt7 deacetylase *in vitro*, Sirt7 inhibitor 97491 (5 μM, MCE, CAT# HY-135899) was used for the first two days. The culture medium was replaced every day to prevent the loss of drug activity.

To induce simultaneous senescence and calcification in HASMCs, cells were cultured in DMEM/F12 medium (1:1) supplemented with 5 mM phosphate (prepared as a 1:2 mixture of NaH_2_PO_4_ and Na_2_HPO_4_, pH 7.4) and 50 μg/mL ascorbic acid. Ang II (0.6 μM, Sigma, CAT# A6778) was added to the culture, and cells were treated for 5 days. The medium was replaced daily to maintain drug activity.

### Measurement of NAD^+^/NADH Ratio

The NAD^+^/NADH ratio was measured using an NAD^+^/NADH detection kit (Beyotime, CAT# S0175) according to the manufacturer's protocol. Briefly, approximately 1×10^6^ cells were added to 300 μL of pre-cooled NAD^+^/NADH extraction buffer and gently pipetted. The mixture was then centrifuged at 12,000 × g, 4 °C for 8 min, and the supernatant was collected for further analysis. To detect total NAD^+^ and NADH, 20 μL of the supernatant was mixed with aldehyde dehydrogenase working solution and added to a 96-well plate, followed by incubation at 37 °C in the dark for 10 min. After incubation, 10 μL of chromogenic agent was added and incubated at 37 °C for 30 min, and the absorbance was measured at 450 nm using a microplate reader. To measure NADH only, NAD^+^ in the supernatant was decomposed by heating at 60 °C. After 30 min, the detection was carried out. The detection method for NADH was the same as for NAD^+^. A standard curve was established by measuring a series of dilutions of NADH standards. The ratio of NAD^+^/NADH was determined by the following equation: ratio= (NAD_total_ - NADH)/NADH. Here, NAD_total_ denoted the amount of total NAD (NAD^+^ NADH); NADH denoted the amount of NADH, both were calculated from the standard curve.

### Western blot

Cells and tissues were lysed using RIPA lysis buffer (Beyotime Biotechnology, CAT# P0013B) containing protease and phosphatase inhibitor (Beyotime Biotechnology, CAT# P1045) for total protein extraction. Western blotting was performed to assess the levels of proteins in tissues or cells, including P16, P21, P53, γH2AX, Rb, p-Chk1, IL-6, IL-1α, TNF-α, Arg1, NQO1, HMOX1, P62, PINK1, Cycline1, Sirt7, RUNX2, BMP2, Osx, Nrf2.

### Detection of Keap1 acetylation by co-immunoprecipitation (Co-IP)

The aorta and cultured HASMCs were lysed in immunoprecipitation (IP) buffer (Beyotime Biotechnology, CAT# P0013) with protease and phosphatase inhibitor (Beyotime Biotechnology, CAT# P1046). After sonication and centrifugation at 13,000 × g for 20 min, the lysate supernatant was incubated overnight at 4 °C with the specified primary antibody, followed by incubation with Protein A/G PLUS-Agarose (Thermo Fisher Scientific, CAT# 78610) at 4 °C for 2 h. The samples were washed five times with cold IP buffer and then resuspended in loading buffer. Cell lysates and immunoprecipitates were denatured in loading buffer at 95 °C for 5 min, and Keap1 acetylation levels were analyzed by Western blotting.

### Statistical analysis

By the analysis and recommendations of the experimental design, all data from this study were analyzed using GraphPad Prism 9.0.0. Continuous data that conformed to normal distribution were expressed as mean ± SEM. Data were compared between two groups using the Student's *t* test. And one-way ANOVA with Turkey post hoc test was performed to compare multiple data sets. Significance was considered when P < 0.05.

## Supplementary Material

Supplementary figures and tables.

## Abbreviations

GLSP: *Ganoderma lucidum* spore powder
GAA: Ganoderic acid A
GAB: Ganoderic acid B
GAC6: Ganoderic acid C6
GAG: Ganoderic acid G
GMT: Ganodermanontriol
SASP: senescence-associated secretory phenotype
HFD: high-fat diet
Rb: Retinoblastoma protein
H2AX: H2A histone family member X
Chk1: Checkpoint kinase1
IL-1β: Interleukin-1β
IL-6: Interleukin-6
TNF-α: Tumor necrosis factor-α
MMP3: Matrix metalloproteinase 3
MMP13: Matrix metalloproteinase 13
ICAM-1: Intercellular adhesion molecule-1
VCAM-1: Vascular cell adhesion molecule-1
IL-1α: Interleukin-1α
NQO1: NAD(P)H quinone dehydrogenase 1
HMOX1: Heme oxygenase 1
P62: Sequestosome 1
PINK1: PTEN induced putative kinase 1
SRA: Type A scavenger receptor
ABCG1: ATP-binding cassette transporter G1
RUNX2: Runt-related transcription factor-2
BMP2: Bone morphogenetic protein 2
ALP: Alkaline phosphatase
Keap1: Kelch like ECH associated protein 1
Nrf2: Nuclear factor erythroid 2-related factor 2
Ang II: Angiotensin II
ROS: Reactive oxygen species
MDA: Malondialdehyde
CAT: Catalase
