# Supplementary material for: GLSP mitigates vascular aging by promoting Sirt7-mediated Keap1 deacetylation and Keap1-Nrf2 dissociation
Source: Theranostics. 2025 Mar 18;15(10):4345–67. doi: 10.7150/thno.110324 (PMC11984382; doi:10.7150/thno.110324)
Supplement: Supplementary file 1 — Supplementary figures and tables. [file thnov15p4345s1.pdf]

GLSP mitigates vascular aging by promoting Sirt7-mediated Keap1 deacetylation and Keap1-Nrf2 dissociation

Yanfei Cheng[1#], Guobin Zheng[2#], Heming Huang[4#], Jingyu Ni[1], Yun Zhao[1], Yuting Sun[1], Yingxin Chang[1], Shangjing Liu[1], Feng He[6], Dan Li[1], Yuying Guo[1], Yaodong Miao[5], Mengxin Xu[1], Dongyue Wang[1], Yunsha Zhang[1], Yunqing Hua[1], Shu Yang[3*], Guanwei Fan[1*], Chuanrui Ma[1*]

[1]First Teaching Hospital of Tianjin University of Traditional Chinese Medicine, National Clinical Research Center for Chinese Medicine Acupuncture and Moxibustion, Tianjin, China

[2]NHC Key Lab of Hormones and Development and Tianjin Key Lab of Metabolic Diseases, Tianjin Medical University Chu Hsien-I Memorial Hospital & Institute of Endocrinology, Tianjin 300134, China

[3]Department of Geriatrics, Peking University Shenzhen Hospital, Shenzhen, China

[4]Department of Geriatrics, Shenzhen People's Hospital (The Second Clinical Medical College, Jinan University; The First Affiliated Hospital, Southern University of Science and Technology), Shenzhen, Guangdong 518020, China.

[5]Second Affiliated Hospital of Tianjin University of Traditional Chinese Medicine, Tianjin, P. R. China

[6]Culture and Industry Research Center of Li Shizhen Traditional Chinese Medicine, Li Shizhen College of Traditional Chinese Medicine, Huanggang Normal University, Huanggang, 438000

#These authors contributed equally to this article.

Correspondence should be addressed to:

Chuanrui Ma, PhD; Guanwei Fan, PhD; Shu Yang, PhD;

First Teaching Hospital of Tianjin University of Traditional Chinese Medicine, National Clinical Research Center for Chinese Medicine Acupuncture and Moxibustion. No.88, Chang Ling Road, Li Qi Zhuang Jie, Xi Qing District, Tianjin, P.R. China;

E-mail: chuanruima2013@mail.nankai.edu.cn; guanwei.fan@tjutcm.edu.cn; yang.shu@szhospital.com;

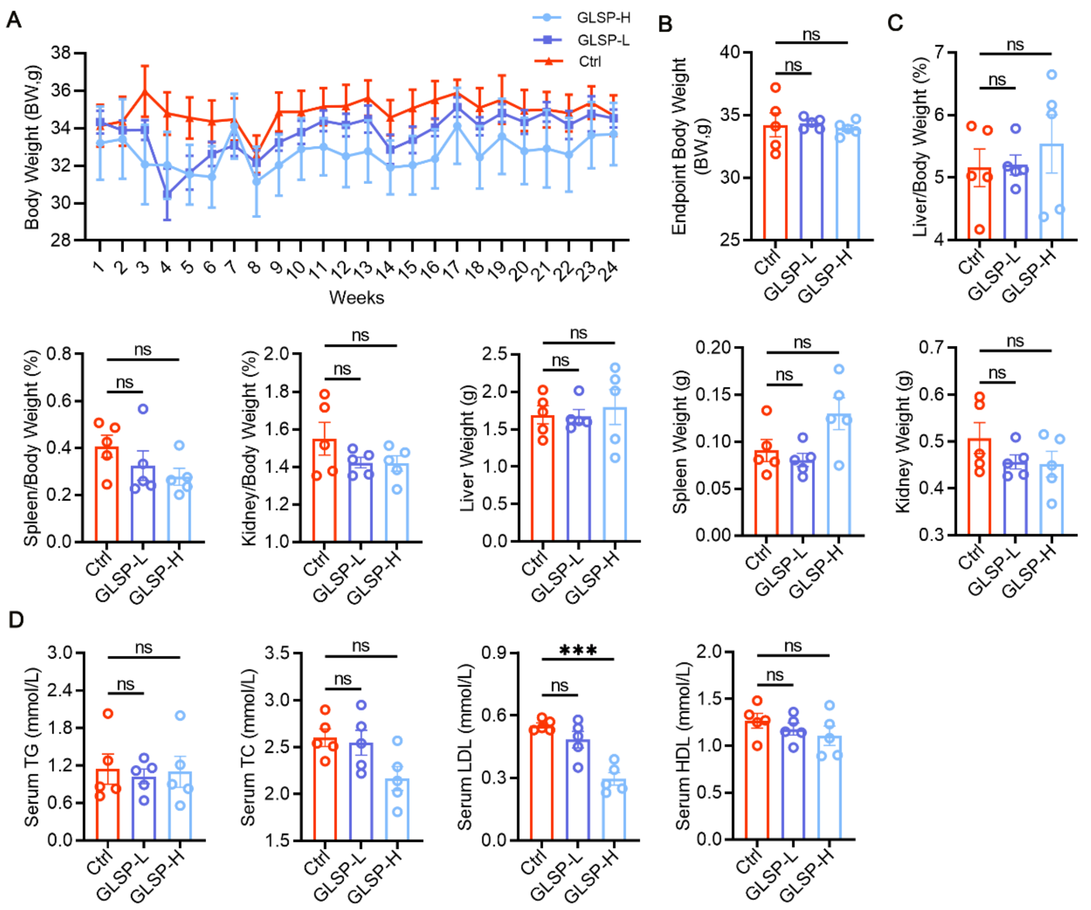

Figure S1. Body weight, organ index, and blood lipid levels of aged mice. (A) Line chart of body weight changes in naturally aged mice during the administration period (n = 5). **(B)** Body weight of mice after the completion of administration (n = 5). **(C)** Liver weight, spleen weight, kidney weight, liver-to-body ratio, kidney-to-body ratio, and spleen-to-body ratio of naturally aged mice (n = 5). **(D)** Levels of TG, TC, LDL and HDL in the serum of naturally aged mice (n = 5). *P<0.05, **P<0.01, ***P<0.001, ****P<0.0001. All experiments were compared with the Ctrl group and error bars denote SEM. Ctrl: Control group.

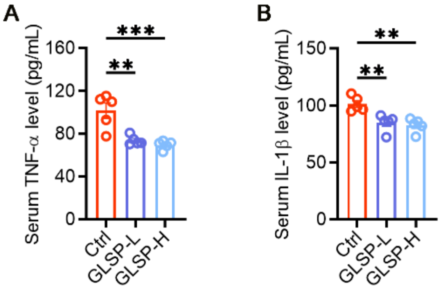

Figure S2. GLSP reduced the expression of inflammatory factors in the serum of naturally aging mice. (A) ELISA was used to detect the expression level of TNF-α in serum (n = 5). **(B)** ELISA was used to detect the expression level of IL-1β in serum (n = 5). *P<0.05, **P<0.01, ***P<0.001, ****P<0.0001. All experiments were compared with the Ctrl group and error bars denote SEM.

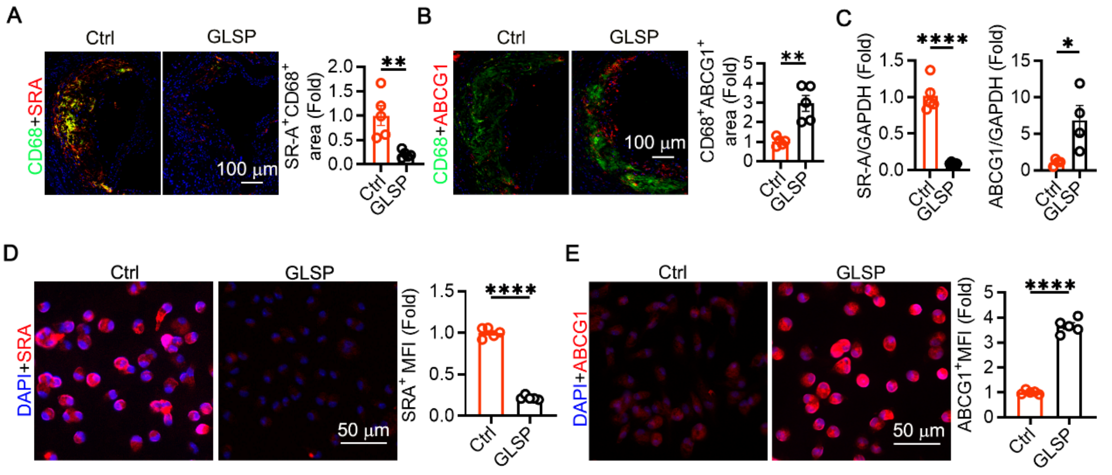

Figure S3. GLSP demonstrated the ability to modulate lipid metabolism in mice with advanced atherosclerosis. (A, B) Immunofluorescence staining revealed changes in SR-A and ABCG1 levels within aortic root plaques (n = 5). **(C)** qRT-PCR was used to measure the expression levels of SR-A and ABCG1 in peritoneal primary macrophages (n = 5). **(D-E)** Immunofluorescence staining showed changes in the expression levels of SR-A and ABCG1 in peritoneal primary macrophages (n = 5). *P<0.05, **P<0.01, ***P<0.001, ****P<0.0001. All experiments were compared with the Ctrl group and error bars denote SEM.

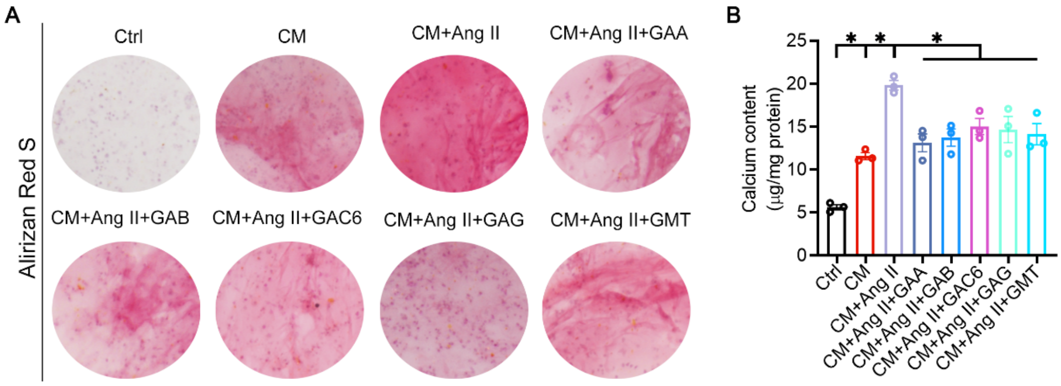

Figure S4. The principal component of GLSP effectively attenuated the calcification levels in a VSMC model associated with aging and calcification. (A) Alizarin red staining in an in vitro model of aging combined with calcification (n = 3). **(B)** Calcium content assessment in an in vitro model of aging combined with calcification (n = 3). *P<0.05, **P<0.01, ***P<0.001, ****P<0.0001. All experiments were compared with the Ctrl group or the CM group or the CM+Ang II group and error bars denote SEM.

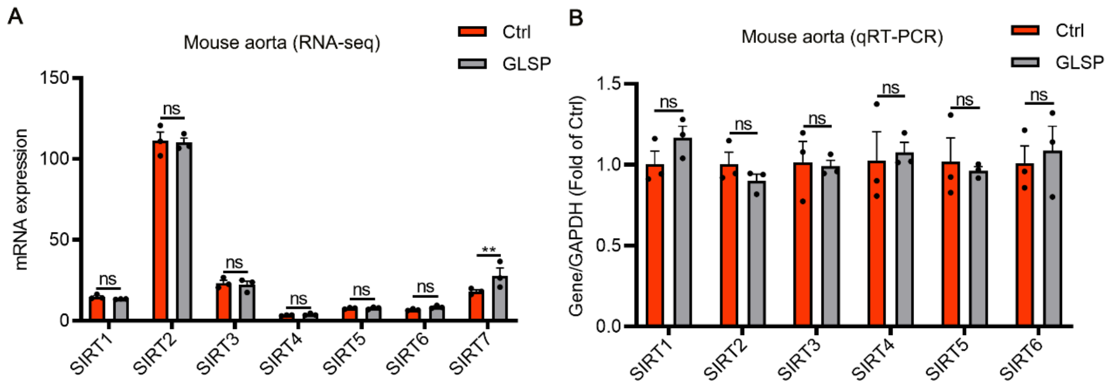

Figure S5. Changes in the expression levels of Sirt family genes. (A) Transcriptome sequencing demonstrated significant alterations in the expression levels of Sirt family genes upon GLSP treatment in this study (n = 3). **(B)** qRT-PCR analysis of the expression of Sirt1-6 in the aorta of aged mice (n = 3). *P<0.05, **P<0.01, ***P<0.001, ****P<0.0001. All experiments were compared with the Ctrl group and error bars denote SEM.

Table S1. the sequences of primers for qRT-PCR analysis

Gene	Forward primer	Reverse primer
Mus P53	GCGTAAACGCTTCGAGATGTT	TTTTTATGGCGGGAAGTAGACTG
Mus P21	CCTGGTGATGTCCGACCTG	CCATGAGCGCATCGCAATC
Mus SR-A	ACGACCCGCCACAATTCTC	CTGGAAGCCTTACTTGAAGGAG
Mus ABCG1	CTTTCCTACTCTGTACCCGAGG	CGGGGCATTCCATTGATAAGG
Mus TNF-α	CCCTCACACTCAGATCATCTTCT	GCTACGACGTGGGCTACAG
Mus Sirt7	CAGGTGTCACGCATCCTGAG	GCCCGTGTAGACAACCAAGT
Mus GAPDH	AGGTCGGTGTGAACGGATTTG	TGTAGACCATGTAGTTGAGGTCA
Mus RUNX2	ATGCTTCATTCGCCTCACAAA	GCACTCACTGACTCGGTTGG
Mus BMP2	GGGACCCGCTGTCTTCTAGT	TCAACTCAAATTCGCTGAGGAC
Mus ALP	CCAACTCTTTTGTGCCAGAGA	GGCTACATTGGTGTTGAGCTTTT
Mus Sirt1	AGAACCACCAAAGCGGAAA	TCCCACAGGAGACAGAAACC
Mus Sirt2	GCCTGGGTTCCCAAAAGGAG	GAGCGGAAGTCAGGGATACC
Mus Sirt3	TGCTACTCATCTTGGGACCT	CACCAGCCTTTCCACACC
Mus Sirt4	GTGGAAGAATAAGAATGAGCGGA	GGCACAAATAACCCCGAGG
Mus Sirt5	CTCCGGGCCGATTCATTTCC	GCGTTCGCAAAACACTTCCG
Mus Sirt6	ATGTCGGTGAATTATGCAGCA	GCTGGAGGACTGCCACATTA

Table S2. Antibodies that used in the manuscript

Antibody	Application	Dilution buffer	Dilution ratio	Number of use	Species
**P16**	IF	1%BSA	1:500	2	Rabbit
**P16**	WB	TBST	1:2000	1	Rabbit
**P21**	IF	1%BSA	1:250	2	Mouse
**P21**	WB	TBST	1:2000	1	Mouse
**P53**	WB	TBST	1:6000	1	Mouse
**TNF-α**	IF	1%BSA	1:250	1	Mouse
**TNF-α**	WB	TBST	1:2000	2	Mouse
**MMP3**	IF	1%BSA	1:500	1	Mouse
**MMP13**	IF	1%BSA	1:250	1	Rabbit
**TOM20**	IF	1%BSA	1:500	1	Mouse
**Arg1**	WB	TBST	1:6000	1	Mouse
**αSMA**	IF	1%BSA	1:500	4	Mouse
**ABCG1**	IF	1%BSA	1:500	1	Rabbit
**Sirt7**	IF	1%BSA	1:500	3	Rabbit
**Sirt7**	WB	TBST	1:2000	8	Rabbit
**HMOX1**	WB	TBST	1:2000	1	Mouse
**NQO1**	WB	TBST	1:6000	1	Mouse
**LC3**	IF	1%BSA	1:500	1	Rabbit
**P62**	IF	1%BSA	1:500	1	Rabbit
**PINK1**	WB	TBST	1:2000	1	Rabbit
**BMP2**	IF	1%BSA	1:500	2	Mouse
**BMP2**	WB	TBST	1:2000	1	Mouse
**IL-6**	WB	TBST	1:1000	2	Rabbit
**Keap1**	WB	TBST	1:2000	8	Mouse
**Nrf2**	WB	TBST	1:6000	2	Rabbit
**IL-1β**	IF	1%BSA	1:500	1	Mouse
**ICAM-1**	IF	1%BSA	1:500	1	Mouse
**VCAM-1**	IF	1%BSA	1:500	1	Mouse
**CD68**	IF	1%BSA	1:500	2	Mouse
**SR-A**	IF	1%BSA	1:500	2	Mouse
**RUNX2**	IF	1%BSA	1:500	2	Mouse
**RUNX2**	WB	TBST	1:1000	1	Mouse
**ALP**	IF	1%BSA	1:500	2	Mouse
**OsX**	WB	TBST	1:1000	1	Mouse
**3-nitrotyrosine**	IF	1%BSA	1:500	1	Rabbit
**4-hydroxynonenal**	IF	1%BSA	1:500	1	Rabbit
**PH3**	IF	1%BSA	1:500	1	Rabbit
**8-Oxoguanine**	IF	1%BSA	1:500	1	Mouse
**γH2AX**	WB	TBST	1:6000	1	Rabbit
**p-Chk1**	WB	TBST	1:6000	1	Rabbit
**CCR2**	Flow Cyt	Standing buffer	1:40	1	Rat
**CD11b**	Flow Cyt	Standing buffer	1:50	1	Rat
**Ly6C**	Flow Cyt	Standing buffer	1:300	1	Rat
